# Rewiring the immune circuit: programming tumor-draining lymph node immunity with nanotechnology

**DOI:** 10.1186/s12951-026-04607-8

**Published:** 2026-05-31

**Authors:** Xu Chen, Meiyan Zou, Nina Li, Zihao Zhou, Rongwei Xu, Weiyao Feng, Zhiyu Guo, Xinyuan Zhao, Li Cui

**Affiliations:** 1 Stomatological Hospital, School of Stomatology, Southern Medical University, Guangzhou, 510280 Guangdong China; 2 School of Dentistry, University of California, Los Angeles, Los Angeles, CA 90095 USA

**Keywords:** Antigen presentation, Sentinel lymph nodes, Tumor-draining lymph nodes, Nanoparticles, Tumor immunotherapy, T cell

## Abstract

Tumor-draining lymph nodes (TDLNs) play a central yet unresolved role in cancer immunotherapy. As obligatory conduits for metastatic dissemination and primary sites of antigen presentation, they represent a biological intersection at which immune activation and immune suppression coexist. Despite this dual role, TDLNs are still more often treated as sites of metastatic risk than as immune niches that can be therapeutically reprogrammed. Nanotechnology provides a means to reprogram these lymph nodes by directing immunomodulatory agents to TDLNs and controlling their intranodal localization, retention and release. In this review, we synthesize nanotechnology-enabled strategies aimed at modulating immune function within TDLNs. Rather than focusing on material composition alone, we articulate general principles that govern lymphatic access, intranodal localization and immune engagement, and examine how these parameters influence therapeutic outcomes. We further discuss key challenges that constrain clinical translation, including the difficulty of achieving sustained and spatially defined immune programming within lymph nodes, the need to balance local immune activation with systemic safety, and the impact of interpatient variability in lymphatic and nodal immune states. Collectively, these considerations frame TDLNs as actionable immunological control points and provide guidance for the rational design of next-generation nanoplatforms capable of supporting durable systemic antitumor immunity.

**Graphical Abstract:**

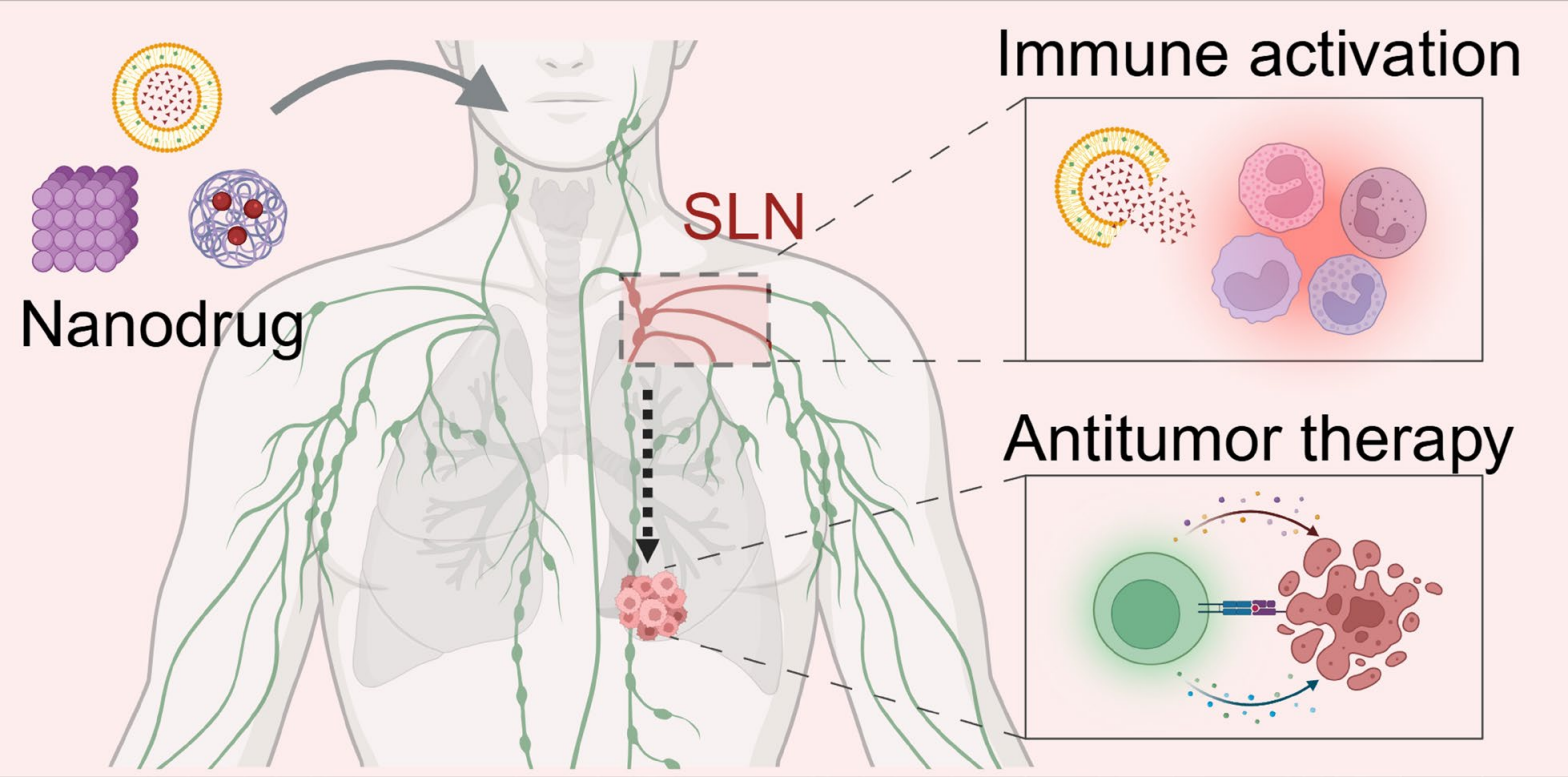

## Introduction

**Fig. 1:**
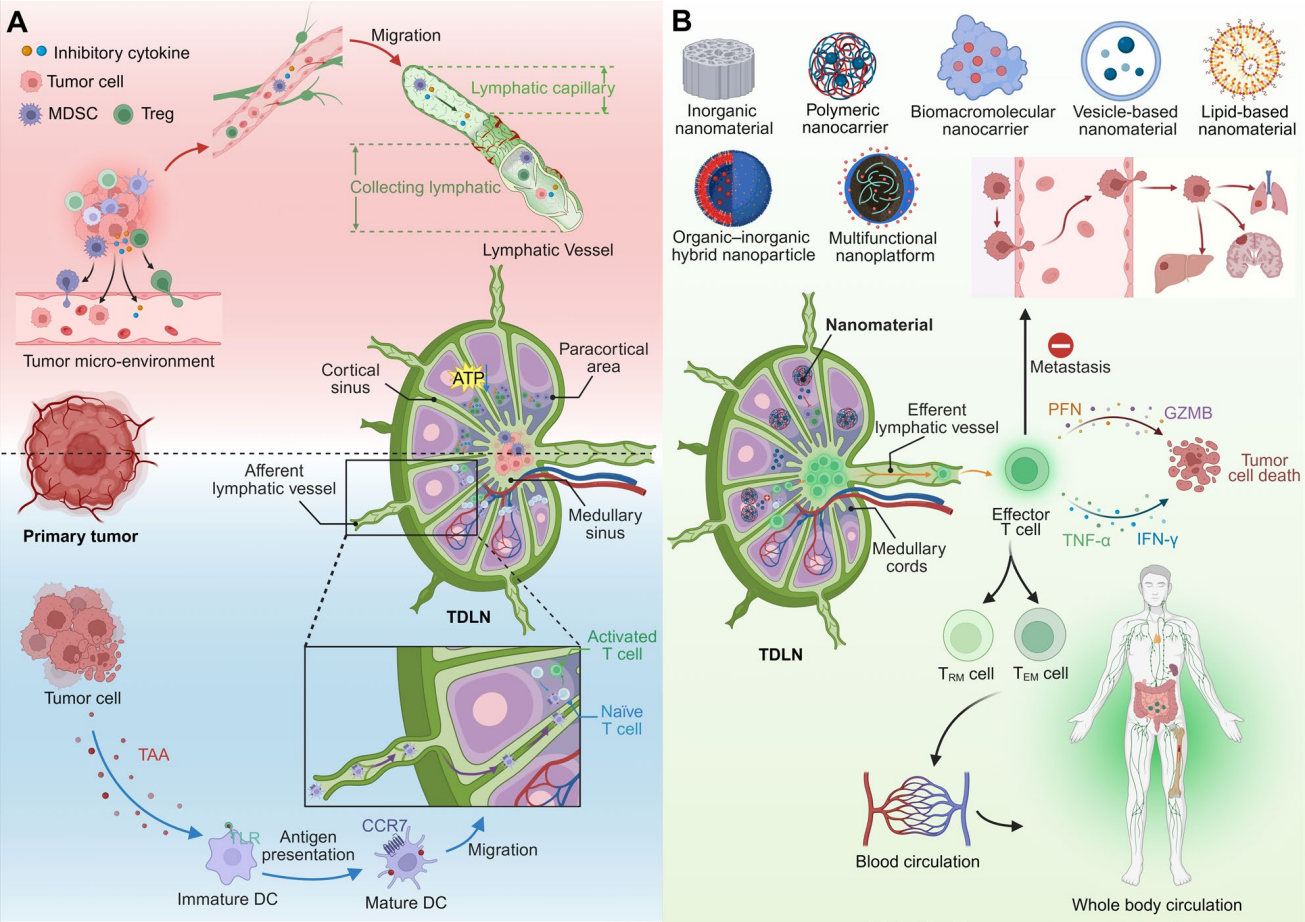
Nanomaterial targeting of TDLNs for immune regulation.** (A)** Tumor-derived signals and migratory immune cells drain from the primary tumor through lymphatic vessels to SLNs and TDLNs. Within TDLNs, dendritic cells capture and present tumor-associated antigens to naïve T cells, positioning these lymph nodes as primary sites for the initiation of adaptive immune responses. At the same time, continuous tumor-derived immunosuppressive cues constrain DC function and T cell activation, resulting in inefficient priming and the establishment of an immunologically restrained nodal niche despite ongoing antigen exposure. **(B)** Nanomaterials designed for lymphatic transport preferentially access and accumulate in TDLNs following administration. Their selective nodal localization reshapes the immune microenvironment within the lymph node, enhancing immune activation and supporting more effective T cell priming and expansion. Activated effector T cells subsequently exit TDLNs and enter systemic circulation, where they mediate antitumor immune responses at primary tumor sites and distal metastatic lesions

Lymph nodes (LNs) are central hubs of the immune and lymphatic systems, where T cells, B cells and dendritic cells (DCs) develop, interact and continuously survey lymph for signs of infection or abnormal cellular growth [1, 2]. In cancer, tumor-draining lymph nodes (TDLNs) broadly refer to the nodal network exposed to tumor-derived antigens, suppressive soluble factors and, during progression, metastatic cells, whereas sentinel lymph nodes (SLNs) represent the first nodal station along tumor lymphatic drainage and are most relevant to early metastatic seeding, sentinel node biopsy and first-station intervention [3]. This distinction is clinically and immunologically important: SLNs may be tumor-free, occultly involved or limited to early metastatic deposits, yet they can already display an immunotolerant landscape and impaired lymph node-resident DC activation [4, 5]. By contrast, broader TDLNs can serve as reservoirs for systemic antitumor T cell responses and are required for effective PD-1/PD-L1 checkpoint therapy, but sustained tumor exposure and nodal involvement may progressively shift them toward immune dysfunction [6, 7]. Extensive clinical and experimental evidence has established that TDLNs and SLNs act as obligatory gateways for tumor cell spread to distant organs [8]. At the same time, these lymph nodes constitute the primary anatomical sites at which tumor-associated antigens (TAAs), released from the tumor microenvironment, are captured, processed and presented to naïve immune cells [9, 10]. This functional duality places TDLNs at the intersection of metastatic dissemination and antitumor immune initiation, endowing them with disproportionate influence over the magnitude and quality of adaptive immune responses **(**Fig. 1A**)**.

This dual role, however, is not fixed across malignancies or disease stages. TDLNs are dynamic immune organs whose cellular composition and suppressive programs vary with tumor type. For example, melanoma- and breast cancer-draining LNs have been reported to show dysfunction of DC subsets and impaired antigen-presenting capacity, whereas cervical cancer-draining LNs can be prominently shaped by macrophage/Treg/checkpoint-associated suppressive programs and pre-metastatic immune remodeling [11, 12]. These tumor-type-specific patterns are further reshaped during tumor progression. In early disease, TDLNs may retain immune-surveillance functions, but sustained tumor exposure, metabolic remodeling and nodal involvement can gradually shift them toward immune suppression [6, 13]. Importantly, this transition may begin before overt nodal metastasis, as premetastatic breast TDLNs already show dynamic B cell and IL-21-producing follicular helper T (Tfh) cell-associated remodeling, indicating that nodal immune reprogramming can precede detectable tumor colonization [14]. Once nodal involvement occurs, immune plasticity may further evolve toward suppression; in head and neck cancer, TDLN neutrophils support antitumor responses in metastasis-free settings but acquire suppressive properties after nodal metastasis [15]. Thus, TDLN-targeted nanotherapies should not assume a fixed immune niche, but should account for cancer-type-specific nodal architecture and stage-dependent transitions from immune surveillance to immune suppression. Across these heterogeneous contexts, the immunological potential of TDLNs is frequently undermined by tumor-induced suppressive programmes, including the accumulation of regulatory immune populations, inhibitory cytokine networks and metabolic constraints that collectively impair effective antigen presentation and T cell priming [16–19]. This biology underlies a long-standing clinical dilemma. Surgical excision of TDLNs can interrupt metastatic progression, yet simultaneously abolishes a critical site of immune activation, thereby limiting the efficacy of immunotherapy [7, 20]. Conversely, preserving TDLNs maintains immune-priming capacity but risks facilitating tumor cell colonization and outgrowth [21, 22]. Therapeutic strategies should therefore move beyond simple lymph node removal and instead combine metastatic control with preservation or restoration of nodal immune function.

In this context, nanotechnology has emerged as a conceptually transformative strategy to address the tumor-lymph node paradox. Because SLNs and broader TDLNs represent distinct tumor–lymphatic contexts, nanodesign should be tailored to the intended scenario. For early-stage SLN-oriented intervention, nanoplatforms may prioritize nodal localization, retention and first-station interception, as illustrated by Titanium nitride nanobipyramids for SLN mapping and photothermal ablation [23]. Magnetic nanoclusters designed for photothermal ablation of tumor sentinel lymph nodes further exemplify energy-responsive elimination of first-station nodal disease [24]. When metastatic SLNs are already established, ligand-guided cytotoxic nanoplatforms, such as hyaluronic acid-conjugated high-density lipoprotein (HDL)-mimic melittin nanoparticles, may be more appropriate for local eradication of metastatic nodal deposits [25]. By contrast, late-stage TDLN-oriented strategies should emphasize immune reprogramming rather than nodal ablation, using nanoparticle-coupled antigen/adjuvant delivery to reactivate immunosuppressed TDLNs [26], adjuvanted nanoparticles to reshape TDLN antitumor immunity [27], or checkpoint-modulating nanocarriers to restore productive T cell function [28]. More broadly, nanoscale materials can exploit physiological lymphatic drainage to preferentially access and accumulate within TDLNs and SLNs following local or systemic administration [29–31]. Such lymphotropic nanocarriers enable the spatial confinement of immune checkpoint inhibitors (ICIs), adjuvants, cytokines and other immunomodulators to immune-active sites, thereby enhancing local immune activation while minimizing systemic toxicity [32–34]. Advances in materials design—including precise control over particle size, surface chemistry and stimulus-responsive release—have further enabled nanoparticles to coordinate immune modulation across the tumor microenvironment and its associated lymphoid tissues, effectively coupling local tumor perturbation with downstream lymph node programming [35, 36] **(**Fig. 1B**)**.

In this review, we systematically synthesize nanomaterial-based strategies that target SLNs and TDLNs as immunological control points for cancer therapy. Rather than organizing these approaches solely by material composition, we focus on how inorganic, polymeric, biomacromolecular, vesicle-based, lipid-based and hybrid nanoplatforms differentially engage lymphatic transport, nodal retention and immune programming. Across these material classes, we highlight how lymphatic homing, nodal retention and stimulus-responsive behavior can be rationally engineered to overcome the limited efficacy and systemic toxicity of conventional immunotherapies. Together, these advances delineate a unifying framework in which lymph node targeting, immune reprogramming and tumor-lymphatic crosstalk are integrated to convert TDLNs from sites of immune suppression and metastatic risk into engines of durable systemic antitumor immunity.

## Inorganic nanomaterials enabling lymphatic targeting and nodal immune priming

Inorganic nanomaterials represent a foundational class of lymph node-targeted immunotherapeutic platforms, distinguished by their controllable size, surface functionality and physicochemical responsiveness [37]. Metallic, metal-compound, silicon-based and carbon-based nanostructures access SLNs and TDLNs through lymphatic transport while enabling functions that are less accessible to purely organic carriers, including photothermal conversion, magnetic hyperthermia, image-guided nodal mapping and localized ablation of metastatic lymph nodes [38, 39]. Thus, the major contribution of inorganic platforms is not simply antigen or adjuvant delivery, but the conversion of physical or physicochemical perturbations into spatially confined immune stimulation along the tumor-lymph node axis.

### Metallic nanomaterials

**Fig. 2:**
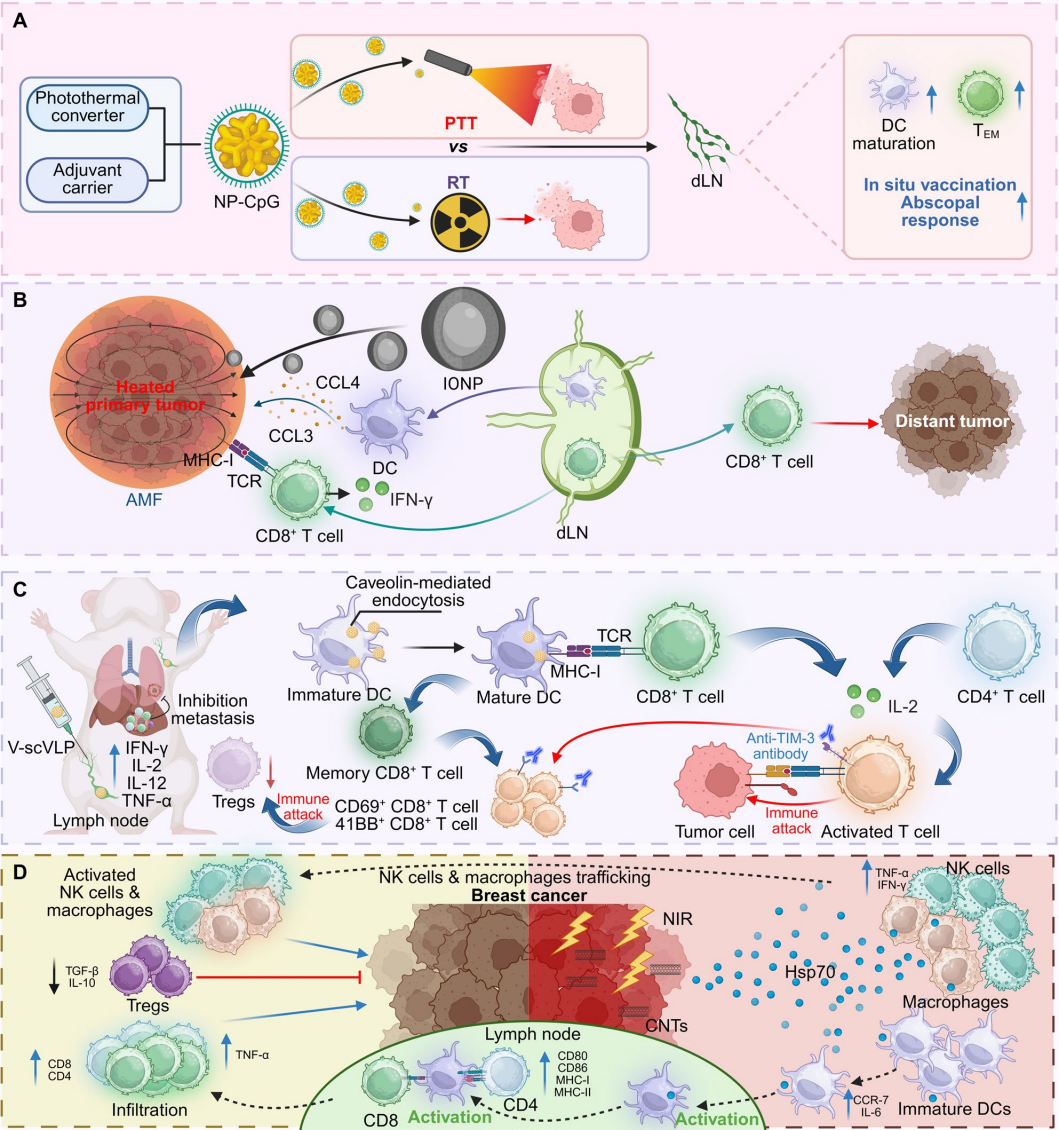
Nanomaterial-enabled hyperthermia as a lymph node-centric amplifier of in situ cancer vaccination.** (A)** Branched gold nanoparticles conjugated with CpG oligonucleotides function as combined photothermal converters and immunoadjuvant carriers. Upon PTT, localized tumor destruction is coupled to enhanced DC maturation in primary and secondary draining lymph nodes, resulting in expansion of effector memory T cells and induction of a systemic in situ vaccination effect with abscopal tumor control. **(B)** Iron oxide nanoparticles activated by an AMF generate controlled hyperthermia that remodels immune signaling along the tumor-lymph node axis. Heating of primary tumors to immunogenic temperatures (~43 °C) promotes DC activation within TDLNs, followed by sustained priming and expansion of cytotoxic CD8^+^ T cells that confer durable, tumor-specific protection, whereas excessive thermal stress abrogates effective immune priming. **(C)** V-scVLPs bearing spike-like topologies enable efficient co-delivery of tumor neoantigens and TLR9 agonists to dendritic cells via caveolin-mediated endocytosis, driving DC maturation, robust CD8^+^ T cell activation, and central memory formation. Concomitant modulation of immune checkpoint signaling, including TIM-3, further enhances antitumor immunity when combined with checkpoint blockade. **(D)** Acid-functionalized multiwalled carbon nanotubes potentiate hyperthermia-induced immunogenic cell death (ICD), characterized by extensive tumor necrosis and Hsp70 exposure, and amplify immune activation within TDLNs. The resulting coordinated recruitment and activation of DCs, CD8^+^ and CD4^+^ T cells, macrophages, and NK cells promote effective tumor eradication and long-term survival. Collectively, these paradigms illustrate how nanomaterial-assisted hyperthermia transforms local tumor ablation into a systemic, lymphoid-amplified antitumor immune response

Metallic nanomaterials, with their tunable size, surface chemistry and plasmonic properties, offer efficient lymphatic trafficking and precise immunomodulatory intervention within TDLNs. These features are particularly evident in gold-based systems, in which ultrasmall particle size supports lymphatic transport of immune agonists, whereas branched architectures provide plasmonic responsiveness and high-capacity oligonucleotide conjugation. For instance, gold nanoparticles of ~5 nm, coated with mixed alkanethiols, act as amphiphilic carriers that adsorb unmodified Toll-like receptor 7 (TLR7) agonists and transport them intact to TDLN after subcutaneous administration. Their nanoscale dimensions enable rapid lymphatic trafficking, where the retained immunostimulant triggers localized activation of antigen-presenting cells (APCs) and amplifies tumor-specific cytotoxic T cell priming. This targeted delivery markedly suppresses the growth of large established tumors and prolongs survival, underscoring the capacity of minimalist gold nanocarriers to concentrate potent immune cues within TDLNs and overcome insufficient responses seen with free ligands [40]. In addition, branched gold nanoparticles carrying cytosine guanine dinucleotide (CpG) oligonucleotides amplify photothermal-induced immunity in lymphoma by simultaneously triggering local tumor destruction and potent lymphoid activation. Upon photothermal therapy (PTT), CpG nanoparticles markedly increased DC maturation in both primary and secondary draining lymph nodes and elevated CD8/CD4 and effector-to-regulatory T cell (Treg) ratios across tumors, yielding a robust systemic vaccination effect [41] **(**Fig. 2A**)**.

### Metal-compound nanomaterials

Metal-compound nanomaterials—including metal oxides, metal chalcogenides, and metal nitrides—offer unique magnetic, plasmonic, and photothermal properties that enable precise manipulation of immune activity within TDLNs. Fe_3_O_4_-CdSe/ZnS multifunctional nanoclusters combine magnetic and fluorescent properties with favorable biocompatibility, allowing dendritic cells to internalize them without significant loss of viability and enabling subsequent imaging, tracking and magnetic separation. Loading lipid A into the hydrophobic shell yields an immunostimulatory nanocluster that augments DC activation and markedly enhances their migration to TDLNs. The resulting amplification of antigen-specific T cell priming underscores the potential of multifunctional nanoclusters to reprogram SLN immunity and strengthen DC-based cancer immunotherapy [42]. Notably, iron oxide nanoparticles activated by an alternating magnetic field generate controlled hyperthermia that remodels immunity within the tumor-lymph node axis. Local heating of B16 tumors to 43 °C triggers robust DC activation in the TDLN, followed by expansion of cytotoxic CD8^+^ T cells that mediate durable, tumor-specific protection against rechallenge. This lymph node-centered priming persists for weeks and scales with primary tumor size, whereas excessive heating abrogates the response [43] **(**Fig. 2B**)**. In addition, superparamagnetic iron oxide nanoparticles (SPIONs) enable highly localized magnetic hyperthermia that reshapes immune activity within tumors and their draining lymph nodes. Intratumoral SPION retention permits controlled heat generation under an alternating magnetic field, inducing spatially confined stress responses and suppressing tumor growth. Immune profiling further revealed enhanced cytotoxic T cell recruitment and coordinated changes in T cell proliferation within both tumors and TDLNs, supporting the immunological impact of spatially confined magnetic hyperthermia [38]. Moreover, 3-aminopropyl-triethoxysilane (APS) magnetic nanoparticles tethered to CD8^+^ T cells preserve cytotoxic function while conferring magnetically controllable trafficking. External magnetic guidance unexpectedly diverted these nano-engineered T cells away from the tumor bed and instead promoted their durable retention within TDLN, where APS- magnetic nanoparticles naturally prolong lymphatic residency. This selective LN sequestration yielded sustained pools of activated tumor-specific T cells despite reduced intratumoral infiltration [44]. Importantly, titanium nitride nanobipyramids enabled multimodal SLN mapping by combining strong plasmonic scattering in both visible and near-infrared windows with efficient lymphatic drainage. Their large anisotropic geometry enhanced optical contrast in bright- and dark-field imaging, allowing precise identification of tumor-draining nodes. Upon 1064-nm irradiation, the nanobipyramids converted NIR-II light into heat with high efficiency, permitting selective photothermal ablation of metastatic lymph nodes. When paired with primary tumor resection, this nodal targeting strategy substantially suppressed downstream pulmonary metastasis [23]. Likewise, hollow titanium nitride nanoshells provide a multifunctional platform that overcomes the limitations of carfilzomib in solid tumors by coupling high-capacity drug loading with intrinsic autophagy inhibition. Their strong near-infrared (NIR)-II photothermal conversion generates synergistic cytotoxicity while promoting deep intratumoral damage. Critically, these nanoshells efficiently drain into TDLN, enabling proteasome inhibition and photothermal ablation within metastatic niches. When integrated with surgical resection, this combined chemo-photothermal strategy markedly suppresses lymphatic dissemination [45].

### Silicon-based nanomaterials

Silicon-based nanomaterials feature exceptional biocompatibility, large surface area, and highly tunable architectures. Their versatile physicochemical properties support efficient cargo loading and structural programmability, enabling broad adaptability across biomedical applications. Virus-like silicon nanoparticles (V-scVLPs) with a spike topological structure efficiently co-deliver hepatocellular carcinoma (HCC)-specific neoantigens and TLR9 agonists to DCs via caveolin-mediated endocytosis. This delivery strategy enhances the activation of lymphoid DC maturation, promoting robust CD8^+^ T cell and central memory T cell responses, which prevent orthotopic HCC tumor growth. Additionally, V-scVLPs trigger a TIM-3-mediated immune checkpoint in tumor-infiltrating CD8^+^ T cells, which can be further enhanced by blocking TIM-3 signaling, resulting in improved antitumor immunity [46] **(**Fig. 2C**)**. In addition, mesoporous silica nanoparticles amplified the immunogenic consequences of local radiotherapy by capturing radiation-released tumor antigens and driving their uptake by dendritic cells. Once internalized, the nanoparticles promoted DC maturation within TDLN, leading to heightened cytotoxic T cell priming and trafficking into both irradiated and distant, non-irradiated lesions. This nanoparticle-augmented antigen relay not only strengthened the abscopal response but also counteracted radiotherapy (RT)-induced Treg enrichment, thereby improving systemic tumor control and sensitizing tumors to PD-1 blockade [47]. Notably, hierarchically engineered silica nanospheres integrate quantum-dot imaging with mRNA delivery to resolve two central barriers in DC immunotherapy: efficient antigen presentation and real-time tracking of DC trafficking to draining lymph nodes. By packaging CEA mRNA, the nanospheres drive abundant antigen expression and robust DC maturation, while embedded near-infrared probes enable precise visualization of DC homing from peripheral tissues into TDLNs. This coordinated nodal targeting elicits potent antigen-specific T cell expansion and cytotoxic activity, resulting in marked suppression of tumor growth [48].

### Carbon-based nanomaterials

Carbon nanoparticles showed strong lymphotropism, efficiently black-staining axillary sentinel and regional nodes to facilitate precise dissection while minimizing unnecessary clearance. When loaded with epirubicin, the nanoparticles not only preserved high-fidelity nodal mapping but also deposited drug directly within metastatic basins, where intranodal accumulation surrounded tumor clusters and induced localized necrosis with fibroblastic remodeling. This dual-function formulation highlights a tractable strategy for nodal-targeted chemotherapy that enhances surgical visualization while delivering cytotoxic payloads selectively within tumor-involved lymphatics [49]. Acid-functionalized multiwalled carbon nanotubes (ox-MWCNTs) potentiate hyperthermia by amplifying both tumor destruction and lymphoid immune activation. Local heating at 43 °C in the presence of ox-MWCNTs triggered extensive necrosis, reduced proliferation, and heightened Hsp70 exposure, generating strong immunogenic signals. This response extended to TDLN, where DC influx and maturation were markedly increased, accompanied by robust recruitment of CD8^+^ and CD4^+^ T cells, macrophages, and NK cells into the tumor. The coordinated activation of local and lymphoid immunity enabled complete tumor eradication and prolonged survival [50] **(**Fig. 2D**)**. In addition, multiwalled carbon nanotubes engineered to co-deliver doxorubicin (DOX) and CpG orchestrate a coordinated photothermal-chemo-immunologic response that reshapes tumor-lymph node immunity in metastatic melanoma. Upon intratumoral administration, the nanocarriers enhance CpG uptake and DC maturation, while NIR-triggered photothermal ablation and local chemotherapy release antigens that expand CD4^+^ and CD8^+^ T cells within tumors, spleen, and draining lymph nodes. This immune activation is reinforced by reprogramming tumor-associated macrophages toward an M1 phenotype and reducing intratumoral Tregs, enabling marked suppression of tumor progression [39].

Inorganic nanomaterials provide functionalities that are difficult to achieve with organic carriers, particularly image-guided nodal mapping and physically induced immune activation through photothermal or magnetic effects. However, these advantages also define important translational limitations. Unlike degradable polymeric or lipid-based systems that can support controlled antigen or adjuvant delivery, many inorganic platforms rely on indirect immunostimulation mediated by tissue stress, antigen release, or danger signaling. As a result, their immune outcomes are highly dependent on stimulus intensity, spatial confinement, and local tissue context. This dependence narrows the therapeutic window, because insufficient stimulation may fail to induce productive immunity, whereas excessive stimulation may disrupt lymph node architecture, impair antigen presentation, or induce unwanted tissue injury.

In addition, relatively persistent nodal retention and limited biodegradability may complicate repeated administration and long-term safety evaluation, particularly when the goal is sustained TDLN immune reprogramming. These features suggest that inorganic nanomaterials may be more suitable for short-term or spatially defined interventions, such as sentinel lymph node mapping, nodal ablation, and perioperative immune priming, rather than prolonged modulation of immunosuppressive TDLNs as standalone platforms. For durable immune reprogramming, they may need to be integrated with degradable carriers, controlled-release systems, or immune cell-targeted components to improve specificity and reduce long-term tissue burden. Therefore, future design should move beyond maximizing lymph node accumulation and instead coordinate intranodal distribution, stimulus dose-response, immune cell engagement, and material clearance according to the intended clinical scenario.

## Polymeric nanocarriers enabling lymph node-directed immune programming

Polymeric nanoparticles have emerged as a central class of biomaterial platforms for targeting SLN and TDLN, owing to their biodegradability, synthetic versatility and capacity for precise immune programming [51, 52]. By integrating lymphatic transport, surface functionalization and controlled payload release, polymer-based systems enable spatially defined delivery of antigens, adjuvants and immunomodulatory cues within SLNs and TDLNs. Diverse polymer architectures—including poly(lactic-co-glycolic acid) (PLGA), poly(ethylene glycol) (PEG)-based systems and other biodegradable polyesters—enable direct nodal delivery, biomimetic antigen transport and tumor-to-lymph node immune relays, thereby coordinating localized immune activation with reduced systemic exposure.

### PLGA-based nanoparticles

PLGA nanocarriers provide a biodegradable and synthetically adaptable platform for lymph node-oriented immune delivery, allowing controlled cargo release, lymphatic access and biomimetic surface engineering to be integrated within a single formulation. When administered via lymphatically permissive routes, PLGA nanocarriers efficiently enter TDLNs and function as adjuvant depots. For instance, PLGA-encapsulated bispecific TLR7/8 agonists preferentially accumulate in TDLNs following subcutaneous injection, where they sustain local innate immune activation, enhance co-stimulatory signaling and promote MHC-I–restricted antigen presentation. These effects support antigen-specific CD8^+^ T cell priming and durable control of tumor growth and systemic metastasis, highlighting the capacity of PLGA-based delivery to reprogramme TDLN immunity through lymphatic enrichment alone [53].

Beyond passive lymphatic access, surface engineering strategies have been employed to enhance APC recognition, prolong antigen availability and preserve tumor-associated antigen cues in PLGA nanoparticles. Tumor cell membrane-coated PLGA nanoparticles retain native adhesion molecules and chemokine receptors, enabling them to interfere with metastasis-promoting tumor-stroma interactions while simultaneously serving as multivalent antigen reservoirs. After intravenous administration, these biomimetic constructs efficiently localize to draining lymph nodes and promote effector T cell responses, illustrating how membrane-functionalized PLGA systems can couple peripheral tumor modulation with nodal antigen presentation [54]. In addition, erythrocyte-membrane-coated PLGA nanovaccines prolong antigen persistence and promote DC engagement through mannose-mediated uptake, redox-cleavable antigen release and MPLA co-delivery. By establishing a sustained antigen depot and improving lymph node trafficking, these nanocarriers delay tumor onset, limit metastatic dissemination and strengthen IFN-γ-dominated cytotoxic immunity [55].

Active exploitation of DC transport pathways further expands the lymph node-targeting repertoire of PLGA nanocarriers. Apolipoprotein E3 (ApoE3)-decorated biomimetic nanoparticles, composed of an R837-loaded PLGA core enveloped by an antigen-bearing phospholipid membrane, enhance ApoE-mediated uptake by DCs and increase nanoparticle accumulation in draining lymph nodes after subcutaneous administration. By improving antigen-adjuvant co-delivery and antigen cross-presentation, this platform increases IFN-γ-producing CD8^+^ T cells and strengthens the response to PD-1 blockade [56]. Notably, PLGA nanocarriers can also influence TDLN immunity indirectly by reprogramming tumor-resident DCs that subsequently migrate to lymph nodes. In this context, a hyaluronic acid-paclitaxel complex first induces immunogenic tumor cell death and generates tumor-associated antigens in situ. Sequential administration of CpG-loaded and IL-10 siRNA-loaded PLGA nanocarriers repolarizes recruited DCs toward a high IL-12/IL-10 profile. These reprogrammed DCs then traffic to TDLNs, amplify Th1-biased cytotoxic responses and establish a tumor-to-TDLN relay that produces pronounced antitumor efficacy even without direct PLGA delivery to lymph nodes [57].

### PEG-based nanoparticles

PEG-based nanoparticles constitute a versatile class of immunotherapeutic platforms that leverage controlled nanoscale assembly, prolonged lymphatic residence and modular payload integration. Beyond their classical role in improving colloidal stability and pharmacokinetics, PEG architectures regulate the persistence, localization and intracellular delivery of immune-active cargos within tumors and their draining lymph nodes. For example, PEG-poly (L-lysine)-poly (L-leucine) (PEG-PLL-PLLeu) polypeptide nanovaccines co-encapsulating Poly I: C, STAT3-silencing siRNA and tumor antigens exemplify this strategy by reversing STAT3-driven DC dysfunction and upregulating CD86, CD40 and IL-12, thereby restoring antigen-specific T cell priming through coordinated antigen, adjuvant and siRNA delivery [58]. PEG nanocarriers have also been used to improve antigen transport and immune cell tracking. Coassembled Trp2/PPa-PEG nanoparticles support antigen loading and DC-based vaccination, while their intrinsic ^64^Cu-chelating capability enables positron emission tomography (PET)-based visualization of DC trafficking to draining lymph nodes, where antigen-specific cytotoxic responses are initiated [59].

In addition to pure PEG-based systems, PEG-containing polymeric hybrid nanoparticles have been widely explored for lymph node-centred immune modulation, particularly where controlled release and regional confinement of potent immune regulators are required. PEG-chitosan nanoparticles encapsulating the TMEM176B modulator BayK8644 fine-tune innate signalling without compromising antigen cross-presentation, because nanoparticle-mediated release avoids the phagosomal dysfunction associated with free BayK8644 while preserving inflammasome-dependent immune activation in TDLNs [60]. Additionally, PEG-poly(lactic acid) (PLA) nanoparticles displaying surface-tethered TLR7/8 agonists confine innate immune activation to tumors and draining lymph nodes, reducing systemic cytokine release while potentiating PD-L1 blockade [61]. In parallel, mPEG-PLA nanoparticles encapsulating the PD-1-targeting peptide P-F4 improve peptide stability and preserve high-affinity PD-1 engagement, resulting in increased cytotoxic effector activity and reduced Treg frequencies as a compact alternative to antibody-based checkpoint inhibition [62].

Beyond serving as a structural component of polymeric carriers, PEGylation also functions as a cross-platform surface-engineering strategy to improve lymphatic trafficking, nodal retention and in vivo stability [63]. For example, PEGylated nanovesicles assembled from endogenous tumor cell membranes enhance lymphatic delivery and immunogenicity of tumor lysate antigens, and their combination with PD-1 blockade supports durable tumor regression and long-term immune memory [64]. PEGylation has also been incorporated into imaging and photothermal nanoplatforms, such as Galectin-1–targeted PEG@MnPb-IR820 nanoparticles achieve selective TDLN retention for dual MRI and near-infrared imaging-guided photothermal ablation, while PEGylated Fe_3_O_4_-graphene nanocomposites combine photothermal tumor destruction with immune activation in TDLNs [65]. These examples indicate that PEGylation can extend beyond polymeric drug delivery to support lymph node-oriented stability, retention and multimodal immune intervention.

### Polyester-based polymeric nanoparticles

**Fig. 3:**
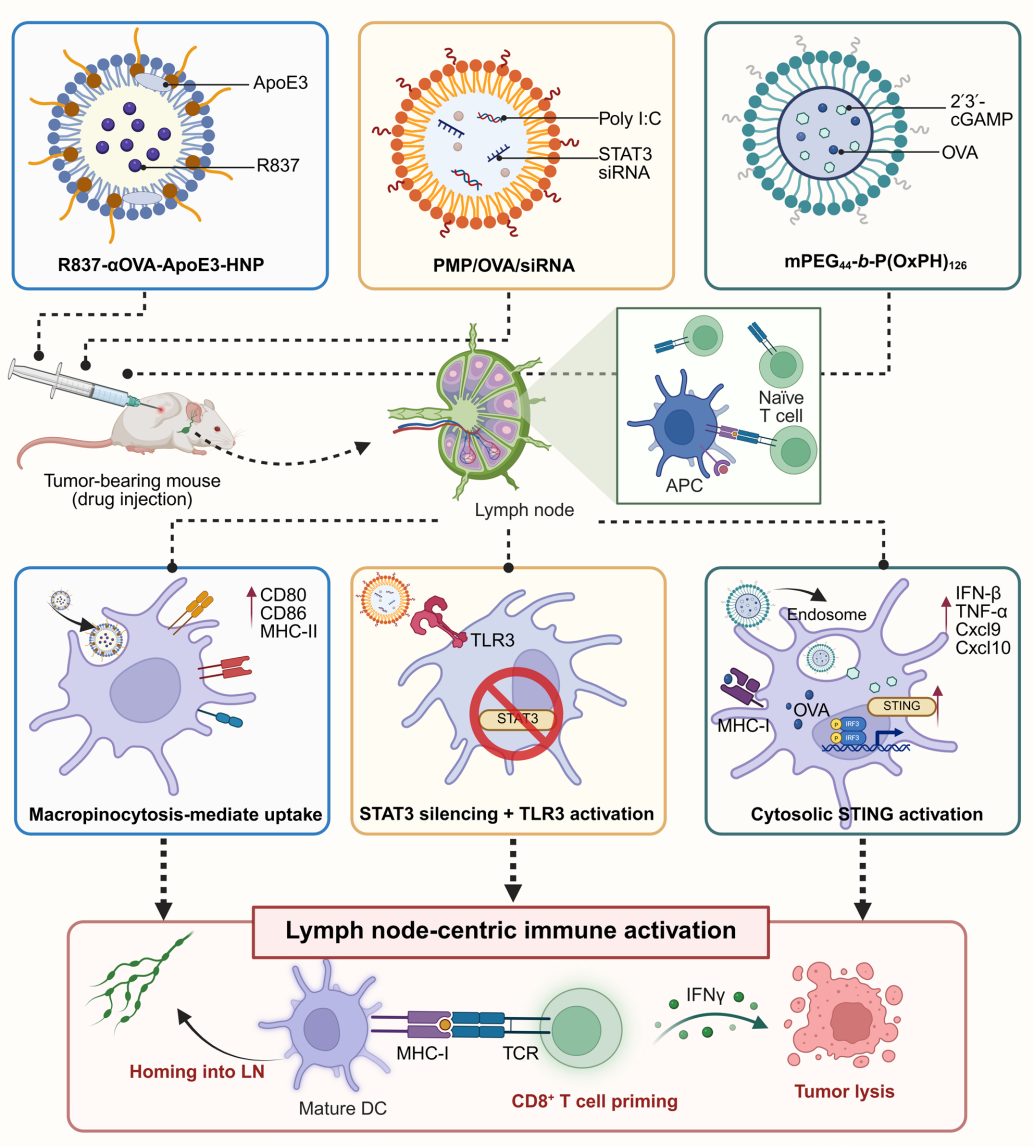
Polymeric nanocarriers enabling lymph node-directed immune programming. A PLGA-based biomimetic nanoparticle (R837‑αOVA‑ApoE3‑HNP) exploits ApoE3‑mediated macropinocytosis to boost antigen/adjuvant uptake, driving DC maturation and cytotoxic T cell expansion. Meanwhile, a PEG‑based polypeptide micelle (PMP/OVA/siRNA) co‑delivers Poly I: C and STAT3‑siRNA to reverse DC dysfunction through combined TLR3 activation and STAT3 silencing. Further, a self‑degradable polyester nanovaccine enables cytosolic release of cGAMP and antigen via pH‑responsive autodegradation, activating the STING pathway and amplifying CD8^+^ T cell priming. Collectively, these strategies spatially concentrate immune activation within TDLNs, synergizing with checkpoint blockade to elicit potent antitumor immunity

Beyond PLGA and PEG-based systems, other biodegradable polyester-based polymeric nanoparticles, such as poly(β-amino ester) (PBAE) and poly(ε-caprolactone) (PCL), expand the polymeric toolbox for lymphatic immune targeting by supporting nucleic acid delivery, cytosolic immune sensing and high-capacity protein antigen loading. Electrostatically self-assembled PBAE/mRNA nanoparticles can be produced at scale with diameters ranging from 200 to 1,000 nm, allowing size-dependent modulation of immune responses following intravenous administration. Particles of approximately 400 nm exhibit optimal efficiency in transfecting circulating monocytes and promoting their differentiation into macrophages and DCs, while CpG co-loading further amplifies adaptive anticancer immunity within TDLNs [66]. Biodegradable PBAE nanovaccines have also been engineered to amplify TDLN-centred cytotoxic immunity through STING activation. A self-degradable PBAE nanovaccine co-delivering 2′3′-cGAMP and antigen rapidly traffics to SLNs following intratumoral injection, where pH-responsive autodegradation facilitates endolysosomal escape and cytosolic release of both the STING agonist and antigen. This design amplifies type I interferon signalling, enhances CD8^+^ T cell priming and improves tumor control while minimizing systemic inflammation associated with free cGAMP [67]. Alongside these nucleic acid- and adjuvant-based strategies, PCL-ovalbumin (OVA) nanovaccines assembled via thiol-disulfide exchange achieve exceptionally high antigen loading (≈ 70–80 wt%) while preserving native protein conformation. Their prolonged retention at the injection site and within TDLNs improves antigen presentation and supports both Th1 and Th2 immune responses, ultimately enhancing tumor resistance [68] **(**Fig. 3**)**.

Polymeric nanocarriers are particularly valuable for TDLN-directed immune programming because their composition, degradation rate, surface chemistry and payload structure can be engineered to control lymphatic transport, antigen persistence and APC activation. Compared with inorganic platforms, their main advantage lies less in physical tumor ablation and more in programmable delivery of antigens, adjuvants and immunomodulators. However, this flexibility also creates translational challenges: small changes in polymer chemistry, particle size, ligand density or release kinetics may substantially alter intranodal distribution, immune cell uptake and therapeutic consistency.

A key design trade-off is persistence versus clearance. Sustained antigen or adjuvant release may enhance T cell priming and immune memory, but excessive nodal retention or repeated dosing may increase the risk of chronic inflammation, off-target innate activation or suppressive feedback. Conversely, rapid degradation may improve safety but weaken immune priming. Different polymer classes therefore require scenario-specific use: PLGA systems are suitable for controlled antigen/adjuvant release but require careful control of degradation and cargo release; PEGylated systems improve stability and lymphatic transport but may reduce cellular uptake or raise concerns related to repeat dosing; PBAE platforms are attractive for nucleic acid delivery and cytosolic immune sensing but require control of cationic toxicity and systemic inflammation. Thus, polymeric nanocarriers should be evaluated not only by lymph node accumulation, but by whether their degradation, release kinetics and immune cell specificity match the intended goal, such as prophylactic vaccination, perioperative priming or therapeutic reprogramming of immunosuppressive TDLNs.

## Biomacromolecular nanocarriers enabling nodal immune priming

Biomacromolecular nanoparticles have emerged as a distinct class of immunotherapeutic platforms that exploit endogenous biological motifs to engage SLNs and TDLNs with high specificity and immunological fidelity [69]. Comprising polysaccharide-, protein/peptide- and nucleic acid-based assemblies, these systems combine intrinsic biocompatibility with lymphatic tropism, receptor recognition and innate immune sensing. By mimicking pathogen-associated structures or leveraging endogenous immune-recognition pathways, macromolecular nanocarriers can concentrate immune activation within nodal niches while reducing systemic toxicity [70].

### Polysaccharide-based nanoparticles

**Fig. 4:**
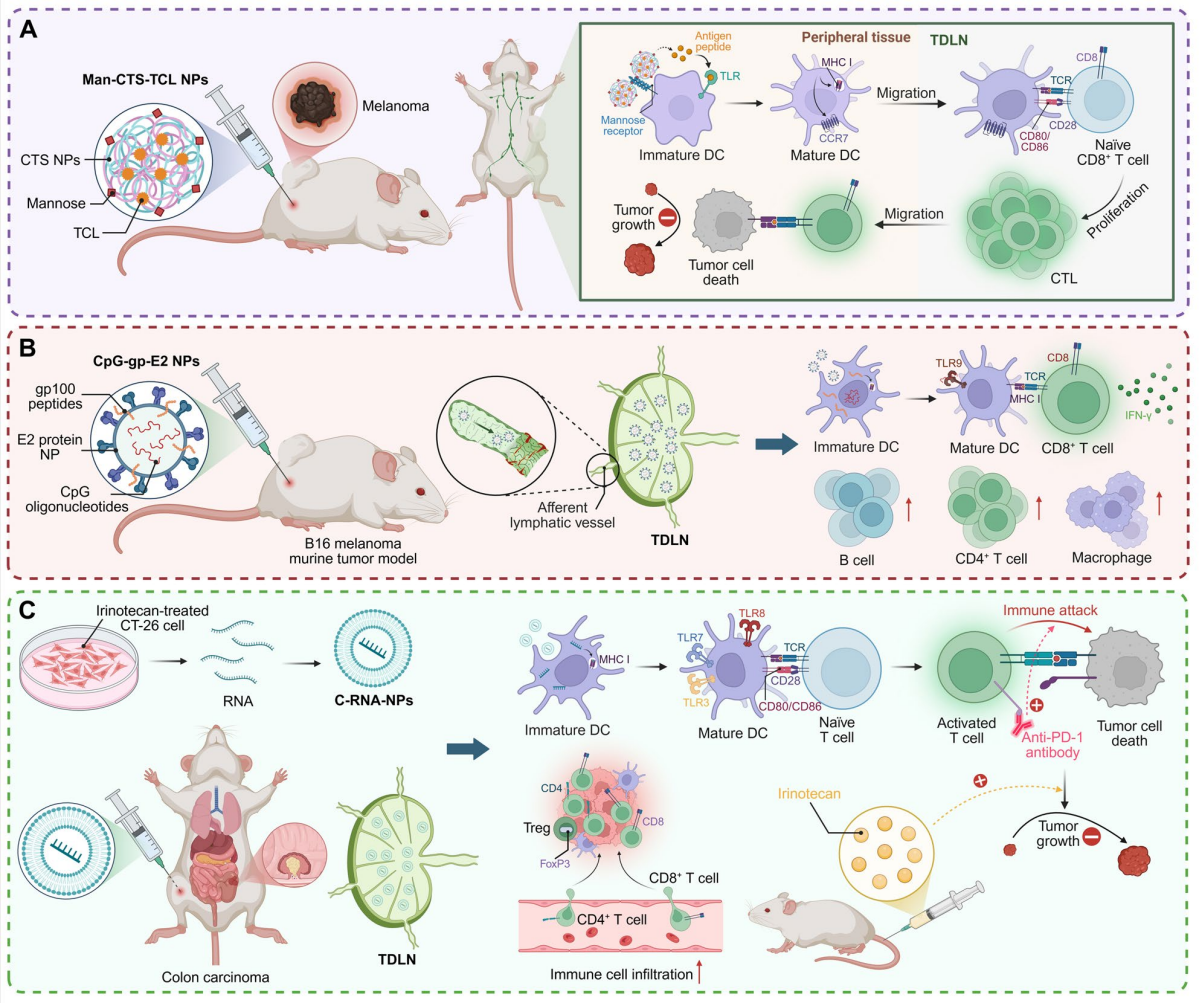
Biomacromolecular nanoparticle-mediated immune priming in TDLNs.** (A)** Mannose-modified chitosan nanoparticles loaded with tumor cell lysates (TCL) are administered and captured by DCs via mannose receptor-mediated recognition. This uptake induces DC maturation and migration to TDLNs, where antigen presentation promotes the priming and expansion of CD8^+^ T cells, leading to cytotoxic T lymphocyte generation and tumor growth suppression. **(B)** Protein-based NPs carrying tumor-associated peptides and CpG oligonucleotides access TDLNs through lymphatic drainage. Local DC activation enhances antigen presentation to CD8^+^ T cells and supports coordinated activation of CD4^+^ T cells, B cells and macrophages, resulting in amplified antitumor immune responses. **(C)** Tumor-derived RNA obtained from irinotecan-treated cancer cells is condensed into C-RNA-NPs and delivered to TDLNs. C-RNA-NPs promote DC maturation and T cell priming, leading to enhanced effector immune cell infiltration and tumor growth suppression. Chemotherapy and PD-1 blockade further enhance the antitumor efficacy of C-RNA-NPs, resulting in strengthened immune responses and improved therapeutic outcomes

Polysaccharide-based nanoparticles leverage intrinsic biocompatibility, lymphatic tropism and carbohydrate-mediated immune recognition to engage TDLNs as central hubs for antitumor immunity. By exploiting carbohydrate-lectin interactions and pathogen-mimetic signaling, these nanomaterials provide a biologically recognizable interface for lymph node-resident APCs while limiting systemic toxicity [71]. Mannose-mediated recognition provides a representative route for directing polysaccharide nanocarriers to nodal DCs. Mannose-decorated chitosan nanoparticles loaded with whole-tumor lysates accumulate in TDLNs after subcutaneous administration and are rapidly internalized by resident DCs, leading to enhanced antigen processing, increased IFN-γ and IL-4 production, and delayed melanoma progression [72] **(**Fig. 4A**)**. Similarly, mannan-decorated polymeric nanovaccines combine optimized lymph node trafficking, preferential CD8α^+^ DC capture, CpG-mediated TLR9 activation and PLA-polyethylenimine (PEI)-enabled endosomal escape, thereby strengthening antigen cross-presentation and durable tumor rejection across multiple models [73].

Beyond ligand-directed DC targeting, microbial polysaccharide-derived nanoparticles demonstrate how intrinsic pathogen-associated molecular patterns can couple efficient lymphatic drainage with innate immune activation. Sugar capsules constructed entirely from microbial polysaccharides form deformable hollow nanostructures that rapidly traffic to TDLNs, where they combine LN-resident DC activation with efficient mRNA delivery for high-level antigen expression. This dual function supports T cell priming and measurable antitumor activity without requiring additional synthetic carrier components [74]. In parallel, yeast cell wall-derived polysaccharide nanoparticles reveal a size-dependent relationship between lymphatic access and immune efficacy. The smallest particles preferentially accumulate in TDLNs and enhance effector immune polarization, while their combination with PD-L1 blockade further improves tumor control, underscoring size-governed lymphatic transport as a key determinant of microbe-derived nanovaccine performance [75].

Natural polysaccharide nanomaterials have also been harnessed to reprogramme immunosuppressive lymph node niches and amplify systemic antitumor immunity [76]. Polysaccharide nanoparticles derived from Astragalus membranaceus engage TDLN circuitry through TLR4-mediated DC activation and promote DC migration to lymph nodes, thereby improving CD4^+^/Treg and CD8^+^/Treg ratios across irradiated and distant lesions and strengthening abscopal responses [77]. Complementing DC-centric approaches, cholesteryl-pullulan nanogels selectively target medullary macrophages within TDLN. Under TLR co-stimulation, these macrophage-targeted nanogels support CD8^+^ T cell cross-priming and enhance tumor control in both prophylactic and therapeutic settings [78]. Importantly, polysaccharide nanocarriers can also transform TDLNs into personalized antigen-processing hubs by capturing endogenous tumor antigens. Mannose-modified stearic acid-grafted chitosan micelles selectively traffic to lymph nodes and bind tumor-derived antigens, facilitating APC uptake and coordinated T cell priming [79]. Likewise, chitosan-tripolyphosphate (TPP) nanoparticles exploit skin-lymphatic connectivity after transcutaneous delivery, accelerating SLN accumulation and promoting both humoral and cellular immunity [80].

### Protein- and peptide-based nanoparticles

Protein- and peptide-based natural macromolecular nanoparticles represent a structurally programmable strategy for LN-centered cancer immunotherapy, owing to their intrinsic biocompatibility, multivalent antigen display and favorable lymphatic transport [81]. In contrast to soluble antigens, these supramolecular assemblies present tumor antigens in a multivalent, virus-mimetic format and exhibit size-dependent drainage that promotes rapid accumulation in SLNs and TDLNs. This property fundamentally reshapes early antigen distribution and immune cell organization within lymph nodes, as exemplified by the eOD-GT8 60-mer protein nanoparticle, which induces markedly enhanced germinal-center activity following subcutaneous administration. Fine-needle aspirate analyses revealed accelerated and amplified antigen-specific B cell and CD4^+^ T cell responses in draining lymph nodes, with subcutaneous delivery generating nearly an order-of-magnitude more germinal-center B cells than intramuscular injection [82].

Building on this principle, self-assembling protein nanoparticles have been engineered to improve antigen density, structural stability and nodal delivery. A BP26 protein nanoparticle displaying tandem MHC II-restricted neoepitopes efficiently traffics to draining lymph nodes, where CpG-augmented APC activation promotes CD4^+^ T cell priming, memory formation and melanoma suppression in stringent B16-F10 models [83]. Modular ferritin nanoparticles engineered via SpyCatcher-SpyTag conjugation similarly enable stable multivalent display of tumor-specific neoantigens. After subcutaneous delivery, these nanocages accumulate in TDLNs and are selectively captured by CD8α^+^ DCs, enhancing cross-presentation and antitumor efficacy, with additional benefit when combined with PD-1 blockade [84]. In parallel, a pyruvate dehydrogenase-based protein nanoparticle co-displaying gp100 antigen and CpG adopts a virus-mimetic architecture that concentrates antigen and adjuvant signals in TDLNs, leading to delayed tumor outgrowth and prolonged survival [85] **(**Fig. 4B**)**.

Peptide-derived natural macromolecular nanostructures further extend lymph node-targeted vaccination by integrating carrier and adjuvant functions within a single assembly. A stapled peptide-based nanovaccine enhances neoantigen peptide stability and cross-presentation while co-delivering nucleic acid adjuvants for toll-like receptor activation, resulting in efficient TDLN targeting, long-term T cell memory and durable protection against tumor recurrence [86]. Beyond antigen delivery alone, an ultrasmall SR-B1-targeted nanovaccine directly delivers tumor antigen peptides to mature DCs in TDLN, overcoming their intrinsically low endocytic capacity and enabling potent antigen presentation with optional CpG co-encapsulation [87]. Notably, a calcium-crosslinked poly(aspartic acid) nanocomplex establishes in situ vaccination by redirecting antigen flow to TDLNs after laser-induced immunogenic tumor destruction, suppressing both primary and distant lesions while inducing durable systemic immunity [88].

### Nucleic acid-based nanoparticles

Nucleic acid-based natural macromolecular nanoparticles constitute an important class of lymph node-oriented immunotherapeutic platforms by combining intrinsic immune sensing with programmable biomimetic delivery. Unlike protein- or peptide-based assemblies that primarily present exogenous antigens, nucleic acid platforms can encode, carry or structurally organize immunostimulatory signals while preferentially accessing TDLNs. Chemotherapy-induced tumor RNA nanoparticles (C-RNA-NPs), generated from the immunogenic transcriptional landscape elicited by ICD-inducing drugs and condensed with protamine, efficiently drain to TDLNs and activate DCs through tumor-derived RNA signals. Compared with non-induced RNA particles, C-RNA-NPs enhance antigen presentation, improve intratumoral CD8^+^ T cell infiltration and elevate the CD8^+^/Treg ratio, thereby restoring cytotoxic immunity and supporting immune checkpoint blockade [89] **(**Fig. 4C**)**. In parallel, an RNA-origami nanostructure functions as both a TLR3-activating scaffold and a precision peptide carrier, enabling uniform antigen loading and localized immune stimulation within TDLNs. This design relieves local immunosuppression and strengthens CD8^+^ T cell responses in combination with PD-1 blockade [90]. Beyond RNA-based systems, a stearic acid-modified CpG oligodeoxynucleotide assembled into a tripod-shaped DNA nanostructure exploits albumin binding to improve plasma stability and lymphatic targeting. Its preferential accumulation in TDLNs induces robust IL-12 production while limiting systemic cytokine leakage, thereby achieving spatially confined TLR9 activation [91].

Biomacromolecular nanocarriers are attractive for TDLN-directed immunotherapy because they exploit endogenous immune-recognition pathways, including lectin binding, pathogen-associated molecular pattern sensing, multivalent antigen display and nucleic acid immune activation. Compared with inorganic or fully synthetic polymeric systems, their strength lies in biological compatibility and immunological familiarity, enabling efficient engagement of DCs, macrophages, B cells and lymph node-resident APCs. However, this biological recognizability also creates a central trade-off: stronger innate sensing may enhance nodal priming, but it may also reduce controllability, increase inflammatory variability or bias responses toward specific immune cell subsets.

Different biomacromolecular platforms therefore require scenario-specific design. Polysaccharide-based systems are useful for carbohydrate–lectin interactions and macrophage/DC uptake, but their activity may vary with carbohydrate structure, molecular weight, purity and receptor expression across TDLNs. Protein- and peptide-based nanoparticles enable defined antigen display and efficient lymph node priming, yet their efficacy depends on antigen density, epitope hierarchy and structural stability. Nucleic acid-based nanostructures can combine antigen encoding with innate immune activation, but their potency must be balanced against nuclease instability and systemic cytokine leakage. Thus, these platforms should not be viewed simply as inherently safer or more “natural” alternatives. Their translational value depends on converting endogenous recognition into predictable immune programming through better control of molecular definition, batch consistency, degradation behavior, receptor-specific targeting and intranodal localization.

## Vesicle-based nanomaterials enabling nodal immune modulation

Vesicle-based nanomaterials, particularly cellular membrane-derived or engineered vesicular systems, have emerged as a distinct class of immunotherapeutic platforms that actively participate in immune regulation rather than serving solely as passive delivery vehicles [92]. By preserving membrane topology and functional biomolecules, these vesicles can directly engage tumor-immune interactions within both tumors and draining lymph nodes [93]. For instance, engineered cellular nanovesicles displaying membrane-bound TRAIL (NVTRAIL) function as active immunomodulators rather than passive carriers. In combination with mitoxantrone, which upregulates GSDME expression, the MTO@NVTRAIL system converts tumor cell apoptosis into pyroptosis, suppressing tumor growth and metastasis while generating pyroptotic microparticles with secondary cytotoxic activity. This inflammatory cell death cascade reshapes immune activation in both tumors and draining lymph nodes, linking vesicle-mediated pyroptosis to systemic antitumor immunity [94]. A pH-responsive vesicular nanovaccine (< 100 nm) co-encapsulating diverse neoantigen peptides with the synergistic adjuvants cGAMP and MPLA further illustrates how vesicular architecture supports lymph node-directed vaccination. Its nanoscale structure promotes lymphatic drainage and resident DC uptake, while pH-responsive cargo release coordinates antigen delivery with STING- and TLR-mediated adjuvant signalling, resulting in enhanced antitumor immunity across multiple tumor models [95]. Vesicle-based nanomaterials are more than passive carriers because their membranes can provide tumor-targeting, death-signaling, and immune-recognition cues. This biomimetic function may help connect tumor killing with nodal immune activation, but it also complicates mechanism attribution, as therapeutic effects may derive from the vesicle membrane, loaded cargo, or secondary inflammation. Therefore, future design should emphasize source-cell selection, membrane composition, batch reproducibility, and controllable immune activation rather than relying broadly on the concept of “natural” vesicle carriers.

## Lipid-based nanomaterials enabling nodal immune modulation

Lipid-based nanomaterials constitute a highly adaptable class of immunotherapeutic platforms for SLN- and TDLN-centred intervention, distinguished by their structural diversity, membrane mimicry and capacity for precise immune modulation. Encompassing liposomes, lipid nanoparticles (LNPs), lipid nanobubbles and nanoemulsions, these systems exploit lymphatic trafficking, lipid-mediated assembly, membrane-compatible cargo loading and externally actuated responses. By integrating antigen delivery, nucleic acid transport, checkpoint modulation or physical triggering, lipid-based platforms coordinate local and systemic antitumor immunity while mitigating off-target toxicity. The following sections delineate how distinct lipid architectures enable complementary strategies for immune priming, reprogramming and amplification across the tumor-SLN/TDLN axis.

### Liposomal nanoparticles

Liposomes remain one of the most versatile lipid-based nanoplatforms for cancer immunotherapy, owing to their bilayer architecture, high cargo flexibility and amenability to lymphatic targeting. By tuning lipid composition, surface functionality and administration routes, liposomal systems can preferentially access TDLNs and spatially confine immunomodulatory signals to key immune-priming sites. Compared with LNPs that are mainly optimized for intracellular nucleic acid delivery, liposomes are particularly suited for membrane-compatible antigen loading, antibody display and regional retention of immunostimulatory cargos. These properties position liposomes as a foundational modality for LN-centred immuno-oncology.

**Fig. 5:**
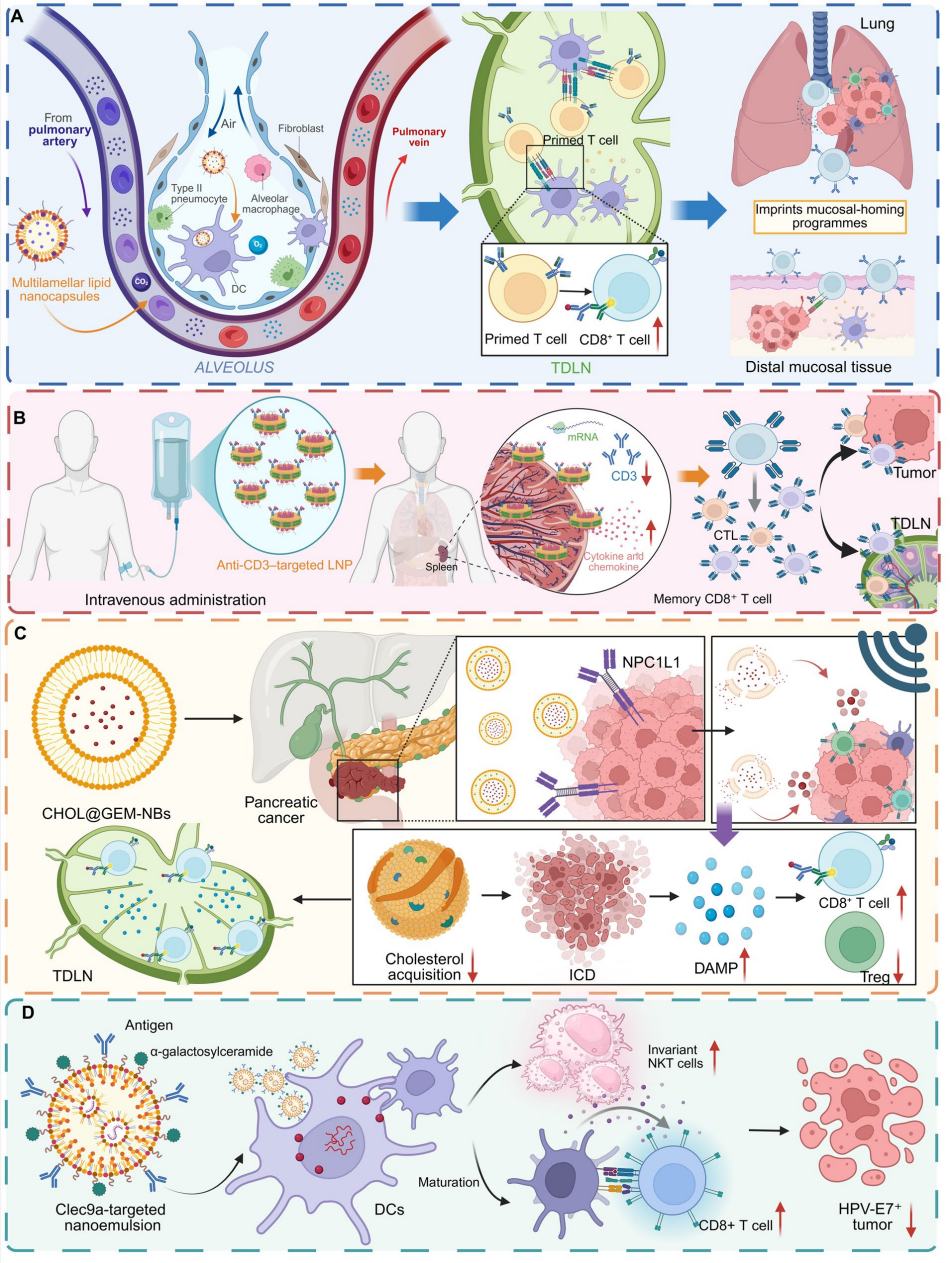
Nanodelivery systems modulate TDLNs to enhance anti-tumor immunity.** (A)** Double-cross-linked multilayer lipid nanocapsules improve antigen stability and delivery efficiency via pulmonary administration. After internalization by lung APCs, they migrate to TDLNs, enhancing cross-presentation and T cell priming, inducing mucosal homing and long-lived effector memory CD8^+^ T cells, and achieving systemic tumor control. **(B)** Anti-CD3-targeted LNPs enable systemic transfection of endogenous T cells after intravenous administration, accumulate in the spleen to mediate mRNA delivery, induce T cell activation and differentiation, and promote their preferential infiltration into tumors and TDLNs. **(C)** CHOL@GEM-NBs target pancreatic lesions and realize ultrasound molecular imaging. Ultrasound-triggered rupture promotes drug release and ICD, thus reprogramming TDLNs. **(D)** Clec9a-targeted nanoemulsions target CD8α^+^ DCs, co-deliver antigen and α-galactosylceramide, activate iNKT cells to synchronize innate and adaptive immunity, and potently activate CD8^+^ T cells for tumor suppression

Several liposomal designs have been developed to enhance TDLN-oriented immunity by improving antigen stability during lymphatic transport or by localizing innate immune stimulation within nodal tissues. Interbilayer-crosslinked multilamellar lipid nanocapsules use reinforced lipid layers to improve antigen stability and delivery following pulmonary administration. After internalization by lung-resident APCs, these nanocapsules traffic to TDLNs, enhance cross-presentation and increase T cell priming compared with soluble formulations. This approach also imprints mucosal-homing programmes and generates durable effector-memory CD8^+^ T cells in pulmonary and distal mucosal tissues, thereby supporting systemic tumor control [96] **(**Fig. 5A**)**. Complementing antigen-focused strategies, NanoCa—a calcium phosphate-containing liposome with an atypical crystalline core and pH-responsive dissolution—functions as a lymphotropic innate immunostimulant. By releasing Ca^2+^ within lysosomes, NanoCa activates a Ca^2+^-calcineurin-NFATc2-PKCβ-IRF3 signaling axis, promoting interferon production and DC maturation. This signaling cascade remodels TDLN immune composition by increasing infiltrating DCs, C1qc^+^ macrophages and CD8^+^ effector T cells while reducing exhausted CD8^+^ T cells and suppressive SPP1^+^ macrophages, thereby strengthening antitumor immune priming and synergising with PD-1 blockade [97].

Beyond vaccine-like functions, liposomes have also been leveraged to localize immunomodulatory antibodies and ICIs to lymphoid niches. A PD-1-antagonist nanoliposome engineered for efficient lymphatic trafficking rapidly accumulates in TDLNs, where regional checkpoint blockade reprograms precursor-exhausted and tissue-resident memory-like CD8^+^ T cell subsets and myeloid-derived suppressor cells, thereby improving intratumoral immune infiltration and therapeutic efficacy relative to non-targeted delivery [28]. Similarly, PEGylated liposomes displaying anti-CD40 antibodies and CpG oligonucleotides confine potent immunostimulatory signals to the tumor and its draining lymph node following intratumoral administration. By sustaining regional APC activation while limiting systemic cytokine dissemination, these nanocarriers preserve antitumor efficacy yet avoid toxicities associated with soluble agonists [98].

### Lipid nanoparticles

In contrast to classical liposomes, LNPs are generally defined by the absence of a stable bilayer-enclosed aqueous lumen and instead rely on ionizable or cationic lipids to form compact nanostructures optimized for intracellular delivery of nucleic acids or immunomodulators. This structural distinction underpins their unique capacity for cytosolic release, endosomal sensing and in situ immune reprogramming. In cancer immunotherapy, LNPs have emerged as a versatile platform for coordinating TDLN priming, intratumoral immune activation and systemic antitumor immunity, often surpassing conventional formulations in both potency and programmability.

A major strength of LNPs lies in their ability to deliver nucleic acids that directly rewire immune cell function. Anti-CD3-targeted LNPs exemplify this paradigm by enabling systemic mRNA transfection of endogenous T cells after intravenous administration. Antibody-guided uptake concentrates these nanoparticles in the spleen, induces transient CD3 downmodulation and promotes effector-memory differentiation, followed by preferential migration of reprogrammed T cells into tumors and TDLNs [99] **(**Fig. 5B**)**. Similarly, ssPalmE-based, pH-responsive LNPs efficiently deliver antigen-encoding plasmid DNA to immune subsets within draining lymph nodes, with preferential uptake by LN-resident macrophages and productive cross-priming by DCs; transient depletion of CD169^+^ macrophages further enhances vaccine efficacy by improving DC access [100]. LNP-mediated nucleic acid delivery has also been used to modulate suppressive metabolic pathways, as shown by cationic lipid-assisted nanoparticles delivering IDO1-silencing siRNA, which dismantle tryptophan catabolism and reinforce oxaliplatin-induced ICD across colorectal and pancreatic tumor models [101]. In parallel, LNPs delivering pDNA or siRNA encoding immune modulators such as OX40L or 2,3-dioxygenase-1 (IDO) achieve robust immune activation, tumor regression and durable memory in melanoma models [102].

Beyond gene delivery, LNPs function as potent immunostimulatory and immunomodulatory platforms through lipid-mediated innate sensing and payload integration. R-1,2-dioleoyl-3-trimethyl-ammonium-propane (R-DOTAP), a defined cationic lipid nanocarrier, couples antigen delivery with endosomal TLR7/9 activation to induce MyD88-dependent type I interferon signaling, lymph node expansion and improved tumor control [103]. LNPs delivering an mRNA cocktail encoding IL-21, IL-7 and 4-1BBL establish a cytokine-enriched intratumoral niche that depends critically on intact TDLNs to coordinate CD8^+^ T cell priming, trafficking and durable memory, indicating that local cytokine expression and nodal immune coordination are functionally linked [104]. Additional design parameters further refine LNP immunobiology. Elasticity-tunable LNPs show that mechanical properties govern macrophage uptake, lymph node transport and STING agonist delivery, with medium-elastic formulations producing superior antitumor outcomes [105]. Advances in lipid chemistry further support translation, as libraries of ionizable 1,2-diesters improve mRNA delivery to spleen and TDLNs while accelerating hepatic clearance and reducing liver toxicity [106].

### Lipid nanobubbles

Distinct from liposomes and LNPs, lipid nanobubbles feature a gas-core architecture that confers ultrasound responsiveness, enabling spatiotemporally controlled payload release and mechanically driven microenvironmental modulation. By integrating external physical energy with lipid-mediated targeting, nanobubbles provide a unique interface between diagnostic imaging, focal therapy and immune reprogramming that is inaccessible to conventional vesicular or nucleic acid-based lipid systems. A representative example is gemcitabine-loaded nanobubbles with a cholesterol component shell (CHOL@GEM-NBs), which exploits the cholesterol transporter Niemann-Pick C1-like 1 (NPC1L1)-mediated uptake for selective accumulation in pancreatic lesions while functioning as a sensitive ultrasound molecular probe. Ultrasound-triggered nanobubble disruption enhances local drug release, suppresses cholesterol acquisition and induces ICD, thereby promoting damage-associated molecular pattern (DAMP) release and and TDLN immune remodeling [107] **(**Fig. 5C**)**. Collectively, this nano-ultrasound strategy couples precise lesion targeting with TDLN-centred immune activation, highlighting lipid nanobubbles as a distinct class of physically actuated immunotherapeutic nanomedicines.

### Nanoemulsions

Nanoemulsions constitute a distinct class of lipid-based immunotherapeutic systems characterized by non-vesicular oil-water architectures and immune engagement mediated primarily through surface functionalization. In contrast to liposomes and LNPs, nanoemulsions lack a stable lipid bilayer or compact lipid-nucleic acid core, instead relying on sustained lymphatic transport and receptor-mediated uptake to enable prolonged immune cell interaction. These features render nanoemulsions well suited for coordinating immune activation within TDLNs, while supporting versatile administration routes, including inhalation. A Clec9a-targeted nanoemulsion exemplifies how immune cell-specific targeting can be leveraged to amplify LN-centred antitumor immunity. By concentrating antigen and α-galactosylceramide within cross-presenting CD8α^+^ DCs, this nanoplatform engages invariant natural killer T (iNKT) cells and natural killer (NK) cells to bridge innate and adaptive activation in TDLNs, resulting in durable tumor suppression in HPV-E7^+^ tumor models [108] **(**Fig. 5D**)**. Extending this strategy, inhalable perfluoro-15-crown-5-ether (PFCE)-C25 nanoemulsions selectively interact with lymphocyte activation gene 3 (LAG-3)-expressing immune cells after pulmonary delivery and undergo efficient lymphatic transport to TDLNs. A single low-dose nebulization induces sustained nodal immune activation, enables ^19^F-MR-based visualization and generates systemic antitumor immunity, prolonged survival and immune memory across multiple lung cancer models, including humanized systems [109] **(**Table 1**)**.

**Table 1:** Overview of lipid-based nanomaterials for targeting and modulating immune responses in TDLN

Type	Carrier characteristics	Mechanism	Immune effects	Applications	Ref.
ICMVs	polyI: C, MPLA	Antigen transport to draining TDLN	Increasing T cell priming	Pulmonary vaccination	[96]
NanoCa	pH-responsive, atypical crystal structure	Triggering interferon through Ca²+-calcineurin-NFATc2-PKCβ-IRF3 pathway	Increasing DC population and CD8^+^ T_eff_ cells	PD-1 blockade	[97]
Peptide-conjugated LPs	Optimal size, serum stability	Lymph node targeting and accumulation in TDLNs	Expansion of resident memory-like CD8^+^T cells	PD-1 blockade	[28]
αCD40/CpG LPs	Surface-conjugated anti-CD40 + CpG	Reducing systemic leakage	Anti-CD40 activation and immune responses	Tumor growth inhibition	[98]
αCD3-LNPs	αCD3-coated, mRNA-loaded LNPs	αCD3 targeting induces CD3e receptor binding	Activation of T cells, CD8^+^ T cells in TDLNs	Tumor immunotherapy	[99]
ssPalmE-LNPs	SS-cleavable, pH-activated ssPalm	Inducing DNA uptake by macrophages and DCs in TDLNs	Activation of macrophages, DCs and T cell responses	Enhancing gene expression and antitumor	[100]
CLANs	Cationic LNPs delivering IDO1 siRNA	Inhibiting IDO1, enhancing DC maturation and T cell infiltration	Enhancing ICD and immunological memory	Enhancing OXA chemotherapy	[101]
LNPs	LNPs delivering pDNA or siRNA	Activating OX40L signaling, inhibits IDO enhances immune cell infiltration	Increasing T cell activation and Treg depletion	Improving local immune activation	[102]
R-DOTAP	R-DOTAP + peptide Ags	Stimulating TLR7/9, MyD88-dependent type I IFN production	Improving Ag-specific CD8^+^ T cells in TDLNs	Synergizing with anti-PD1	[103]
Triplet LNP	LNPs delivering mRNA encoding 4-1BBL	IL-21, IL-7 and 4-1BBL to enhance granzyme B and IFN-γ production	Increasing CD8^+^ T cells, granzyme B and IFN-γ in SLN	Long-term immunological memory	[104]
Lipo-NPs	Elasticity-tunable Lipo-NPs	Activation of macrophages and TILs	Activation of macrophages and TILs	ICB synergy and vaccine design	[105]
1,2-diester LNPs	1,2-diesters with carboxyl-containing alkyl chains	Enhancing protein expression in TDLNs	Enhancing antigen expression in TDLNs, CD8^+^ T cell activation	mRNA vaccines	[106]
CHOL@GEM-NBs	Cholesterol shell, NPC1L1 targeting	Cholesterol uptake modulation via NPC1L1, restoration cholesterol flow	Increasing CD8^+^ T cell and cytokine secretion	Enhancing drug delivery	[107]
TNE	αGC and antigen-loaded TNE, Clec9a targeting	Enhancing cross-priming and recruitment of mature DCs	Enhancing antigen-specific CD8^+^ T cell priming	Long-term tumor suppression	[108]
PFCE-C25 nanoemulsions	Inhalable nanoemulsions targeting LAG-3	Enhancing lymphatic transport	Activation of DCs	ICI	[109]

Lipid-based nanomaterials are clinically familiar and structurally versatile platforms for nodal immune modulation, but their effects depend strongly on lipid architecture and immune context. Liposomes are better suited for regional retention, antigen display, and localized delivery of antibodies or innate agonists, whereas LNPs are more powerful for cytosolic nucacid delivery and transient immune cell reprogramming. Lipid nanobubbles and nanoemulsions further expand this class by enabling ultrasound-triggered release, imaging, or route-specific lymphatic transport. Thus, lipid systems should not be treated as a uniform category, because their immune functions are highly architecture-dependent.

A key limitation is that lipid carriers are not immunologically inert. Ionizable, cationic, or adjuvant-like lipids can affect innate sensing, macrophage uptake, cytokine induction, and organ distribution independently of the intended payload. This may enhance nodal priming when properly confined, but can also increase systemic inflammation, liver/spleen exposure, or cytokine leakage if spatial control is incomplete. Therefore, effective design should move beyond maximizing TDLN accumulation and instead coordinate lipid composition, particle mechanics, administration route, release kinetics, and repeat-dose safety with the intended clinical task. This framework may help distinguish lipid platforms that merely reach TDLNs from those that can reproducibly and safely reprogram nodal immunity.

## Organic-inorganic hybrid nanoparticles enabling nodal immune modulation

Organic-inorganic hybrid nanoparticles have emerged as a powerful class of immunotherapeutic platforms that integrate the physicochemical advantages of inorganic materials with the biological functionality of organic components to modulate tumor-lymph node immune circuits. By combining high imaging contrast, catalytic or photothermal activity with antigen delivery, immune sensing or microenvironment remodelling, these hybrids enable precise navigation to TDLNs or SLNs while simultaneously amplifying immunogenic signals. A defining strength of this materials class lies in its ability to couple local tumor destruction with secondary lymph node immune programming, thereby converting focal interventions into systemic antitumor immunity.

**Fig. 6:**
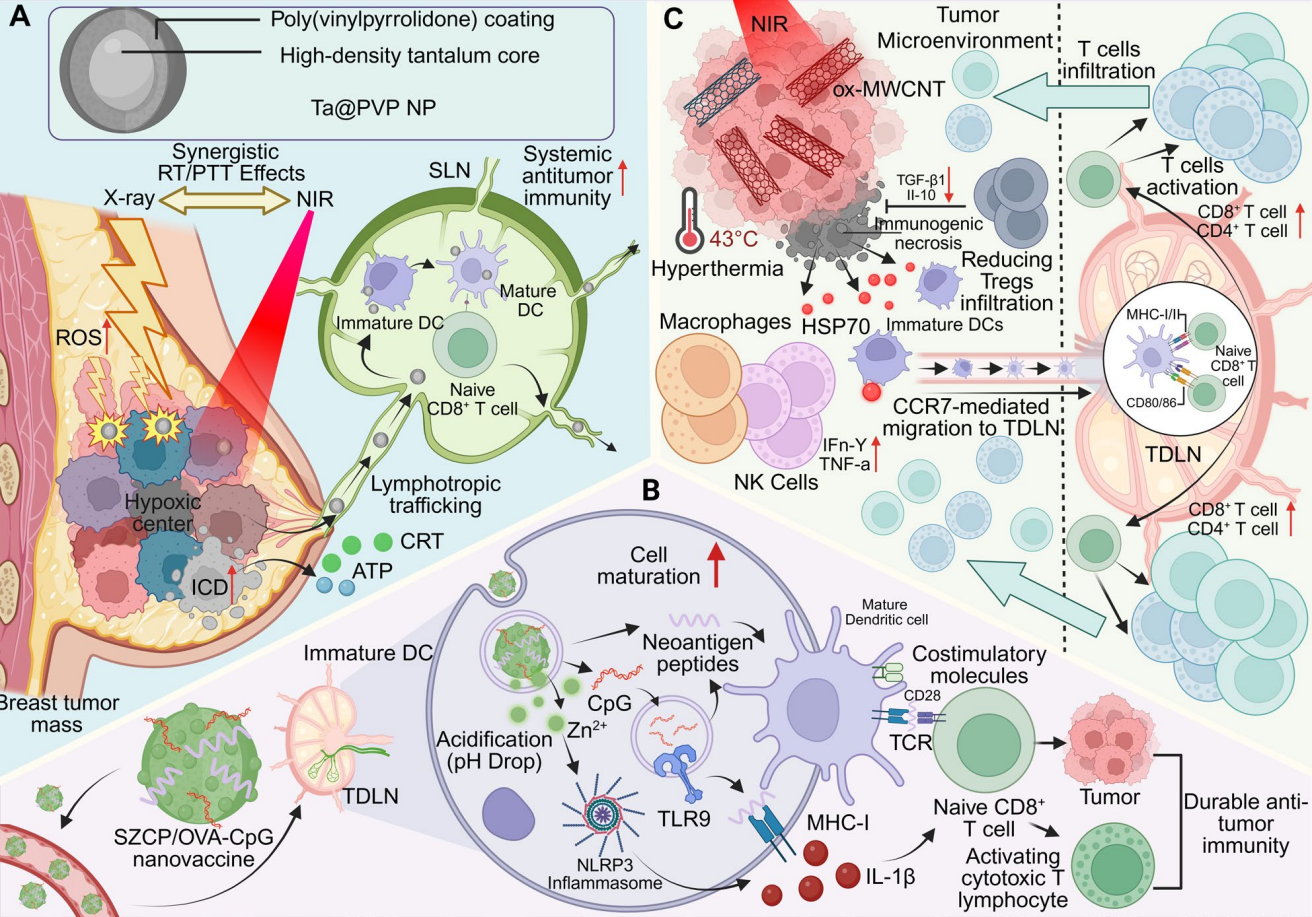
Mechanisms of hybrid nanomaterials in modulating tumor-lymph node immune circuits for enhanced cancer immunotherapy.** (A)** Poly (vinylpyrrolidone)-coated tantalum nanoparticles targeting tumors and SLNs, with high X-ray attenuation for radiosensitization, lymphotropic trafficking for TDLN accumulation, and enhanced photothermal/radiotherapeutic effects upon NIR irradiation. **(B)** Zinc-calcium phosphate nanovaccines loaded with neoantigen peptides and CpG, promoting TDLN-specific immune activation through inflammasome activation, DC maturation, and the upregulation of MHC-I and IL-1β production. **(C)** Oxidized multiwalled carbon nanotubes utilized for hyperthermia-based tumor destruction, leading to enhanced immune activation in TDLNs, including the recruitment of CD8^+^ T cells, macrophages, and NK cells, and improving long-term tumor control

Several hybrid systems exploit inorganic cores to enhance tumor eradication while maintaining access to lymphatic basins. For example, poly(vinylpyrrolidone)-coated tantalum nanoparticles synchronously target breast tumors and metastatic sentinel nodes; their high X-ray attenuation enables radiosensitization of hypoxic primary lesions, while lymphotropic trafficking permits CT- and photoacoustic-visualized accumulation in SLNs. Upon NIR irradiation, combined photothermal-radiotherapeutic effects amplify ICD and trigger downstream immune activation in draining nodes [110] **(**Fig. 6A**)**. Related multifunctional designs further extend this tumor-SLN coupling, including ^131^I-M@HI nanoparticles, in which albumin-based Mn(III) porphyrin assemblies catalytically relieve hypoxia and potentiate radiotherapy while eradicating SLN metastases [111], and CuS-DOX-Nd/FA nanoplatforms that integrate NIR-II imaging with chemo-photothermal therapy to suppress both primary tumors and metastatic nodes [112]. Hollow mesoporous carbon spheres functionalized with PVP (HMCS-PVP) similarly achieve long-retaining SLN targeting, combining high-contrast photoacoustic/photothermal imaging with DOX-mediated chemotherapy to eliminate lymphatic metastases [113].

Beyond physical tumor ablation, hybrid nanoparticles have also been designed to organize antigen/adjuvant delivery and localize innate immune stimulation within TDLNs. Sericin-coordinated zinc-calcium phosphate nanovaccines efficiently deliver neoantigen peptides and CpG into draining lymph nodes, where Zn^2+^ release links inorganic ion signaling with inflammasome activation and IL-1β secretion, supporting durable neoantigen-specific immunity [114] **(**Fig. 6B**)**. Silica-based systems further demonstrate how inorganic scaffolds can confine antigen/CpG stimulation to TDLN-resident APCs while suppressing systemic cytokine leakage. CpG-loaded mesoporous silica nanoparticles cloaked with bacterial outer membrane vesicles (OMVs) additionally incorporate microbial membrane cues to amplify innate sensing, promote M1 macrophage polarization and metabolically reprogramme TDLN T cells to alleviate exhaustion [115]. Parallel inorganic-organic antigen hybrids, such as flower-like OVA-Cu nanostructures, exploit pH-responsive antigen release and macropinocytosis-driven uptake to improve intranodal antigen processing and antitumor immunity [116].

Hybrid nanomaterials have also been leveraged to rewire suppressive tumor and lymph node microenvironments through catalytic, metabolic or structural remodeling. ox-MWCNTs demonstrate that nanotube-mediated hyperthermia can couple local tumor destruction with downstream lymph node immune activation. Local heating at 43 °C induces immunogenic tumor necrosis and Hsp70 exposure, which propagate to TDLNs as broad immune cell recruitment and ultimately enable durable tumour control [50] **(**Fig. 6C**)**. Similarly, more complex nanotube-based platforms integrate physical tumor ablation with targeted immunomodulation. Multiwalled carbon nanotubes co-delivering DOX and CpG orchestrate a coordinated photothermal-chemo-immunologic response, in which NIR-triggered ablation and local chemotherapy promote antigen release while CpG reinforces immune activation across tumor and TDLN compartments, illustrating how combinatorial nanotube systems leverage TDLN-centred immune remodeling to suppress aggressive disease [39]. Gold nanoparticles coated with glycol chitosan establish an immunofunctional photoacoustic readout of SLN integrity by mapping immune cell trafficking patterns disrupted by metastasis, offering a non-invasive diagnostic complement to immunotherapy [117]. In parallel, immunomodulatory hybrid assemblies such as quercetin-iron nanoassemblages suppress PD-L1 signaling while ferrying tumor antigens to TDLNs, metal-polyphenol-wrapped hyaluronidase nanocomplexes remodel extracellular matrix (ECM) stiffness to enhance tumor-TDLN immune crosstalk, and nanoparticle-conjugated TLR7/8 agonists spatially confine innate activation to sentinel nodes to avoid systemic toxicity [118].

Organic-inorganic hybrid nanoparticles are valuable because they integrate complementary functions that are difficult to achieve with single-material systems, including imaging, photothermal or catalytic activity, antigen delivery and innate immune stimulation. This makes them particularly useful for linking local tumor destruction with SLN/TDLN immune activation. However, their multifunctionality also creates a central challenge: when several modules are combined, it becomes difficult to define which component is essential for nodal immune priming, systemic antitumor immunity or treatment-related toxicity. Immune activation may result from photothermal damage, antigen release, adjuvant delivery, inorganic ion signaling or inflammatory tissue injury, and the relative contribution of each mechanism may differ across tumor types and disease stages.

Therefore, hybrid platforms should be evaluated not only by overall antitumor efficacy, but also by mechanistic necessity, design economy and translational feasibility. Compared with single-material systems, they may provide better spatiotemporal coordination of tumor killing and lymph node immune programming, but they also face greater challenges in reproducibility, scale-up, long-term clearance and regulatory classification. Future development should move from simply accumulating functions within one particle toward modular designs in which each component has a defined role, measurable contribution and clinically justifiable benefit.

## Multifunctional nanoplatforms for tumor-lymph node immune programming

A growing body of work has established multifunctional nanoplatforms as an integrative strategy for coordinating therapeutic delivery, physical tumor perturbation and immune modulation across the tumor–lymph node axis. Rather than acting as passive carriers, these nanoplatforms are designed to load, protect and spatially coordinate diverse functional modules—including antigens, adjuvants, nucleic acids, chemotherapeutics and photo-/sono-responsive agents—while exploiting lymphatic trafficking to engage TDLNs/SLNs. In contrast to single-function systems, their main value lies in linking local tumor intervention with downstream nodal immune programming, thereby enabling focal treatment to support systemic antitumor immunity.

Several nanoplatforms harness physical or catalytic tumor injury to amplify ICD and downstream lymph node activation. Photothermal and photodynamic systems based on gold nanorods, mesoporous silica or hybrid metal-semiconductor architectures exemplify this approach. CXC chemokine receptor type 4 (CXCR4)-targeted AuNRs-E5 use photothermal stress to induce immunogenic tumor perturbation and reshape immune activity in both tumors and TDLNs, while reducing Tregs and polarizing macrophages toward an M1 phenotype [119]. Related multifunctional designs, including AuNR@mSiO_2_@DOX-CuS and CuS-DOX-Nd/FA nanoplatforms, integrate chemo-photothermal or photodynamic effects with controlled drug release and image guidance to suppress primary tumors and metastatic dissemination while sustaining systemic immune activation [112]. CaO_2_-based CaO_2_@Curcumin@ZIF-Cu (CCZC) nanoparticles further couple chemodynamic therapy and Ca^2+^ overload to release tumor antigens and inflammatory cues, driving DC maturation in TDLNs and potent cytotoxic T-lymphocyte responses [120]. Oxygen-enriching nanodroplets (PCL@O_2_), when combined with PD-1 blockade after incomplete ablation, similarly enhance ICD and reinforce TDLN-dependent immune memory, illustrating how physical tumor modulation can be translated into durable immune control [121].

Beyond local cytotoxicity, many nanoplatforms are explicitly engineered to optimize antigen delivery and adjuvant activity within lymph nodes. β−1,3-glucan-functionalized aluminium nanovaccines exploit Dectin-1-mediated uptake and aluminium adjuvanticity to activate Syk-Raf1 signaling and potentiate T cell priming in draining lymph nodes, while pullulan nanogel-long peptide antigen vaccines reshape early LN activation dynamics to amplify T cell receptor (TCR)-engineered T cell therapies, achieving durable clinical responses without lymphodepletion [122]. Antigen-focused platforms further include sericin-coordinated Zn/Ca phosphate nanovaccines, MnO_2_-based self-assembling LN vaccines that resolve the granularity paradox, and biodegradable nanoparticles co-delivering poly(I: C), R848 and CCL20 to recondition both tumor and TDLN immune compartments [123]. Additional strategies directly engage innate immune sensing, as exemplified by IAHA-LaP/siPTPN6 nanoparticles that triple-amplify STING signaling in tumor-associated DCs [124], nanoparticle-conjugated TLR7/8 agonists that spatially confine innate activation to sentinel nodes [125], and yeast microcapsule-based Au/Pt composites that combine chemodynamic and photothermal therapy with immune adjuvanticity [126]. Collectively, these systems demonstrate how nanoplatforms can synchronise antigen delivery, innate activation and lymph node-centred T cell priming.

A further layer of sophistication is introduced by nanoplatforms that remodel the tumor or lymphatic microenvironment to facilitate immune trafficking and persistence. Mannosylated chitosan nanoparticles delivered via dissolving microneedle patches exploit skin-resident DCs to drive robust LN maturation [127], while quercetin-iron nanoassemblages suppress PD-L1 signaling, normalize stromal stiffness and ferry tumor antigens to TDLNs to establish durable immune memory [118]. Metal-polyphenol-wrapped hyaluronidase nanocomplexes achieve remotely triggered ECM degradation, strengthening tumor-TDLN immune crosstalk [128], whereas expansile polymeric nanoparticles carrying paclitaxel enhance pH-responsive drug release in TDLNs, suppress local tumor growth, reduce axillary metastasis, and provide a regionally focused strategy for immunotherapy and metastatic control [129]. Platforms explicitly targeting lymphatic disease include hyaluronic acid (HA)-conjugated HDL-mimetic nanoparticles loaded with melittin, which migrate from tumors into SLNs to eradicate nodal metastases [25], and glycol-chitosan-coated gold nanoparticles that provide photoacoustic mapping of immune cell trafficking to non-invasively detect metastatic SLNs [117]. Moreover, pathogen-mimetic systems—such as *Escherichia coli* (*E. coli*)-inspired gold nanorods—combine microbial immune cues with photothermal ablation to programme TDLN immunity and confer resistance to metastatic rechallenge [130] **(**Table 2**)**.

**Table 2:** Multifunctional nanoplatforms for TDLN immune programming

Type	Core functionality	Immune effects	Mechanism	Therapeutic approach	Ref.
AuNRs-E5	CXCR4 antagonist peptide E5 conjugated to AuNRs	Enhancing DC maturation, CD8^+^ T cell infiltration	CXCR4-targeted, ER stress induction via photothermal effects	TNBC photothermal immunotherapy	[119]
CuS-DOX-Nd/FA NPs	Nd-DTPA molecular cluster for NIR II imaging	lymph node tracking and TME modulation	EPR targeting for efficient DOX release and CuS-mediated PTT	Chemo-/photothermal therapy	[112]
PCL@O2	Ce6 as sonosensitizer, PFH as O2 reservoir	Enhancing ICD, increasing T cell infiltration	Inducing ROS generation and calreticulin exposure	Post-iRFA therapy	[121]
CPBG-Al@OVA	Aluminum hydroxide functionalized with β−1,3-glucan	Potent CD8^+^ T cell activation	Activation of Syk and Raf1 signaling pathways	Tumor lysate encapsulation	[122]
Biodegradable polymeric nanoparticles	Delivery of poly (I: C), R848, and CCL20	Enhancing antitumor effects	Poly (I: C) and R848 activate TLR3 and TLR7, MIP3α recruits immune cell	Co-delivery with therapeutic peptide vaccination	[123]
IAHA-LaP/siPTPN6 NPs	La3^+^, cGAMP, and PTPN6 siRNA co-delivery	Enhancing DC maturation, increasing IFNβ secretion	Enhancing cGAMP-mediated STING activation	TDLN immune modulation	[124]
MCS NPs + BCG-PSN	MCS + BCG-PSN and transdermal delivery	Enhancing DC maturation and immune responses	Upregulation co-stimulatory molecules	DC maturation in TDLNs	[127]
QFN	PTT, antigen capture and delivery	Improving T cell infiltration	Inhibiting PD-L1 via JAK2/STAT3 signaling	PTT, TME remodeling	[118]
PPFH	Haase, DFO	Reducing immunosuppression	PPFH disassembly, HAase releaing, ECM remodeling	Tumor ECM modulation	[128]
Pax-eNP	pH-responsive swelling, lymphotropic delivery	Decreasing lymph node metastasis	Enhancing intranodal drug deposition, migration to TDLNs	Locoregional chemotherapy delivery	[129]
MLT-HA-HPPS	Melittin-loaded, HA-conjugated phospholipid nanoparticles	Reducing SLN metastasis	Cytotoxicity through melittin delivery	Metastatic prevention	[25]
GC-AuNPs	USPA imaging	/	Altering spatial distribution in metastatic SLNs	Non-invasive imaging of micrometastases	[117]
ECA	*E. coli* membrane proteins, adhesion proteins	Enhancing T cell immunity	ECA-mediated immune cell activation in TDLNs	Primary tumor treatment	[130]

Multifunctional nanoplatforms are attractive because they can coordinate tumor destruction, antigen release, innate immune activation, lymphatic trafficking and TDLN/SLN immune priming within one system. This design is useful when antitumor immunity requires sequential events, such as ICD induction, antigen capture, APC activation and T cell expansion. However, multifunctionality should not be assumed to indicate therapeutic superiority. When multiple modules are combined, the observed benefit may be driven mainly by one dominant component, such as photothermal injury, chemotherapy or adjuvant exposure, rather than by true synergy among all modules.

Therefore, these platforms should be evaluated not only by overall antitumor efficacy, but also by mechanistic necessity and design economy. Component-deletion studies, dose-matched controls and immune cell-specific readouts are needed to determine whether each module is indispensable. Although highly integrated systems may improve spatiotemporal coordination across the tumor-lymph node axis, they also increase manufacturing complexity, batch variability, regulatory burden and uncertainty regarding long-term biodistribution. Future development should move from simply adding functions toward defining which modules are necessary, synergistic and clinically justifiable.

## Cross-platform immune mechanisms and innate immune gatekeepers

### Convergent antigen delivery, APC engagement and T cell priming

Despite differences in composition and functional design, nanoplatforms that effectively modulate TDLNs tend to converge on a shared sequence of immune events. This process usually begins with increased antigen availability in draining lymph nodes, which can be achieved through antigen-capturing inorganic matrices [47], biomimetic polymeric antigen depots [55], pathogen-like nanovaccines [73], protein-based nanovaccines [83], pulmonary lipid nanocapsules [96] or hybrid antigen scaffolds [116]. These antigenic inputs are then delivered to, retained near or captured by APCs through membrane-derived interfaces [64], carbohydrate-mediated recognition [72], ApoE-assisted uptake [56], multivalent protein display [84] and receptor-targeted lipid assemblies [108]. In parallel, localized innate immune stimulation is introduced through TLR7/8 [53] or TLR9 [91] agonists, STING agonist delivery [67, 95], inflammasome-associated ion signaling [114] and pathogen-mimetic motifs derived from microbial polysaccharides [74] or bacterial membrane components [115]. Through these coordinated processes, distinct material platforms establish a convergent antigen-APC-innate sensing axis that supports antigen processing, DC maturation and early T cell priming within TDLNs.

Once this upstream axis is established, a common downstream immune pattern emerges across polymeric, biomacromolecular, vesicular, lipid-based, hybrid and multifunctional systems. Improved TDLN priming enhances antigen presentation and DC activation [58, 89, 97], promotes antigen-specific CD8^+^ T cell expansion and effector or memory T cell formation [50, 85, 94, 104] and reshapes suppressive immune states, including Treg enrichment, T cell exhaustion, suppressive macrophage polarization and myeloid-derived suppressor cell accumulation [28, 77, 97, 115]. In platforms combined with checkpoint blockade, this reconditioned nodal response further improves PD-1/PD-L1 therapeutic efficacy by replenishing or reactivating the cytotoxic T cell pools required for checkpoint responsiveness [97, 103, 121]. Thus, DC maturation, CD8^+^ T cell expansion and checkpoint sensitization are not isolated findings restricted to individual material classes, but recurrent outputs of coordinated antigen availability, APC engagement and innate immune activation within the tumor-draining lymph node circuit.

### Macrophages as bidirectional regulators of TDLN-targeted nanomaterials

Although DCs are usually emphasized as the main APCs for T cell priming, macrophages form an equally important interface between lymph-borne nanomaterials and the nodal immune environment. After TDLN-targeted nanomaterials enter lymph nodes, their biological effects are not determined solely by their accumulation or delivery efficiency, but also by how they are recognized and processed by the local innate immune system [131, 132]. The subcapsular and medullary sinuses of LNs are enriched with macrophages, and lymph-borne nanoparticles are often first intercepted, internalized, or processed in these regions [133–135]. Thus, macrophages may serve not only as sites of particle deposition and antigen handling, but also as active participants in local immune activation. Cancer-related studies have shown that glycol chitosan-coated gold nanoparticles can accumulate in regional LNs and deliver tumor antigens to LN-resident macrophages, suggesting that macrophage-rich lymphatic sinuses represent an important processing interface for nanoparticles after lymph node entry [136]. Cholesteryl pullulan nanogels further demonstrate that macrophage uptake can be converted into a therapeutic mechanism: after subcutaneous administration, these nanogels are transported to draining lymph nodes and preferentially engulfed by medullary macrophages; in combination with TLR stimulation, they promote antitumor immune responses [78]. This macrophage interface becomes more functionally important when nanomaterials carry innate immune agonists. Polymeric nanoparticles loaded with the TLR7 ligand resiquimod can deliver immunostimulatory signals to macrophages and dendritic cells in draining lymph nodes [137]. L17-F05 mRNA-LNPs further induce a macrophage-centered type I interferon response in draining lymph nodes; depletion of lymph node macrophages reduces APC maturation and antitumor activity, indicating that macrophages can directly contribute to therapeutic efficacy [135]. In addition, small yeast cell wall nanoparticles more efficiently accumulate in TDLNs and remodel the immune microenvironment of both tumors and TDLNs. Their microbe-derived β-glucan/chitin-rich structures also suggest the involvement of myeloid innate immune sensing in this process [75]. However, macrophage engagement is not necessarily beneficial. mRNA-LNP cancer vaccines can increase macrophage-derived itaconate in activated ipsilateral lymph nodes, whereas targeting macrophage Irg1 improves antigen presentation, T cell function, and vaccine efficacy, indicating that lymph node macrophages may also limit antitumor responses through metabolic immunosuppressive feedback [138]. Therefore, macrophages should be viewed as bidirectional regulators of TDLN-targeted nanomaterials: they may enhance antigen handling, local innate immune activation, and antitumor immune amplification, but they may also contribute to particle sequestration, off-target phagocytosis, or suppressive feedback.

### Complement-mediated localization and immune amplification

Complement provides a soluble recognition system through which nanoparticle surface chemistry can be interpreted before or alongside cellular uptake within lymph nodes [139]. During lymphatic transport and lymph node entry, this recognition can alter particle localization, retention and immunostimulatory activity in a surface-dependent manne. A classic study of lymph node-targeted nanoparticles showed that ultrasmall nanoparticles can enter draining lymph nodes via lymphatic transport and activate the complement cascade through their surface chemistry, generating local danger signals; the induced immune responses were dependent on both particle size and complement activation [140]. Hydrogel nanoparticles fabricated using particle replication in nonwetting templates (PRINT) technology can also serve as lymph node-targeting delivery carriers and induce complement activation, as indicated by increased conversion of C3 to C3a [141]. Beyond direct complement activation, complement can also regulate the spatial distribution of nanoparticles within lymph nodes. Studies of glycosylated protein nanoparticle immunogens have shown that surface glycans can be recognized by mannose-binding lectin and subsequently promote particle trafficking to lymph node follicles and germinal centers through a complement-dependent pathway, thereby enhancing antibody responses [142]. Follow-up work further demonstrated that this MBL-complement axis can regulate follicular localization across multiple glycosylated protein nanoparticle platforms, suggesting that complement may serve as a common mechanism governing intranodal homing and immunogenicity of nanoparticles [143]. Together, these lymph node-targeting studies provide a mechanistic basis for considering complement in the context of TDLN-targeted nanomaterials. Therefore, complement should not be viewed only as a source of peripheral inflammation or toxicity, but also act as an innate immune factor that shapes particle localization, local danger signaling, and immune amplification within lymphatic tissues.

## Challenges and future perspectives

Despite rapid advances in nanomaterials engineered to target TDLNs and SLNs, translation into routine clinical immuno-oncology remains constrained by a set of interlinked biological and practical barriers. A fundamental challenge in TDLN-targeted nanomedicine lies in the intrinsic trade-off between efficient lymphatic entry and sustained intranodal retention. Physicochemical features that favour lymphatic drainage—such as small size, hydrophilicity and low adhesive interactions—often promote rapid transit through lymph nodes, whereas prolonged nodal residence typically requires increased cellular or extracellular engagement that compromises lymphatic access [144, 145]. As a result, many nanoplatforms either reach TDLNs transiently without meaningful immune programming or exhibit excessive retention at upstream injection sites. A promising solution is the development of stage-adaptive nanomaterials that decouple lymphatic transport from nodal anchoring. In such systems, nanoparticles are engineered to adopt a lymph-permissive state during interstitial transport and subsequently undergo environment-triggered transformation within TDLNs—through pH, redox or enzyme-responsive mechanisms—to expose adhesion motifs or immunologically active interfaces. This two-step design paradigm enables efficient lymphatic delivery followed by spatially confined immune modulation within nodal niches.

Although lymph node accumulation is frequently used as a primary performance metric, delivery to TDLNs alone does not ensure effective immune activation. Consistent with lymph node vaccine delivery studies, effective nodal targeting is shaped not only by lymphatic drainage, but also by administration route, lymph node architecture, interstitial transport barriers and carrier properties such as size, hydrophilicity, charge and ligand design [146]. TDLNs are highly compartmentalized organs in which immune outcomes are dictated by precise spatial and cellular routing, including access to subcapsular sinus macrophages, cross-presenting DC subsets, follicular regions and T cell-rich paracortical zones [133, 147]. Nanomaterial distribution across these compartments is strongly governed by particle size and surface chemistry. Larger particles, particularly those in the 500–2000 nm range, are less efficient at free lymphatic drainage and more often reach LNs through uptake by migratory DCs from peripheral tissues, whereas smaller particles can drain directly to LNs and interact with LN-resident APCs [140, 148]. After lymphatic entry, particles first encounter the subcapsular sinus, where CD169^+^ subcapsular sinus macrophages can intercept nanovaccines and limit their access to deeper follicular regions [134]. Among freely draining particles, 20–30 nm nanoparticles more effectively penetrate the deep cortex or paracortex, increasing contact with LN-resident DCs and T cell zones [149, 150], whereas 50–100 nm particles can be retained more persistently within follicular DC networks, favoring antigen persistence and B cell responses [151]. Surface chemistry further tunes intranodal trafficking: charge can affect deep cortical transport, while mannose-rich surfaces may promote follicular localization through mannose-binding lectin- and complement-dependent pathways [143]. Together, these findings indicate that intranodal localization is not a passive consequence of LN accumulation, but a design-dependent determinant of immune cell engagement. Nanomaterials intended for macrophage interception, DC/T cell priming or follicular antigen persistence should therefore be optimized with distinct size ranges, surface charges and ligand chemistries. More broadly, advanced delivery-system design should align the intended biological target, local microenvironmental cues and carrier properties to improve cell- or compartment-specific selectivity while limiting off-target effects [152].

Effective T cell priming in TDLNs depends not only on antigen delivery but also on the temporal dynamics and intracellular fate of antigen presentation [6, 153]. Excessively transient antigen exposure may result in incomplete priming, whereas prolonged or uncontrolled persistence risks tolerance induction or T cell exhaustion [154]. Moreover, nanomaterials can differentially bias MHC class I versus class II presentation pathways, with profound consequences for cytotoxic immunity [155]. Future nanoplatforms should therefore enable programmable antigen availability, integrating controlled release kinetics with tailored intracellular trafficking. Strategies that combine an initial antigen pulse with sustained low-level presentation, or that selectively promote cross-presentation through endosomal escape mechanisms restricted to nodal environments, may optimize both the magnitude and durability of antitumor T cell responses while minimizing systemic inflammation.

Potent immune activation within TDLNs inevitably raises concerns regarding off-target inflammation, immune-related adverse events and long-term tissue perturbation, particularly for platforms incorporating strong innate immune agonists or persistent inorganic components [156]. The central safety challenge is to balance sufficient nodal immune stimulation with spatial confinement and temporal control. In this context, the trade-off between local and systemic administration is not absolute, but depends on whether immune activation can be restricted to the tumor-TDLN axis without systemic inflammatory spillover. Local administration can increase tumor-TDLN exposure and reduce the initial systemic burden, yet it does not automatically ensure immune confinement, because soluble or rapidly released agonists may enter lymph-blood recirculation and cause systemic cytokine leakage. For example, soluble cyclic di-GMP (cdGMP) after subcutaneous injection showed limited draining-LN retention and rapid systemic dissemination, whereas nanoparticulate cdGMP redirected STING activation toward draining LNs and reduced systemic exposure [157]. Conversely, systemic administration may be necessary for deep, multifocal or metastatic lesions, but it inevitably broadens exposure of circulating immune cells and liver/spleen mononuclear phagocyte compartments, as shown by systemically delivered STING nanoparticles that improved cGAMP pharmacokinetics but still induced hepatic and splenic immune activation [158]. Thus, the key issue is not whether local delivery is safer than systemic delivery, but how to preserve productive LN priming while avoiding cytokine leakage and off-target immune activation. Retention-oriented and release-controlled strategies, including albumin hitchhiking, depot-based release, anchoring approaches, lymph node-restricted pro-agonists and locally activatable immunomodulators, may help confine immune activation to the tumor-TDLN axis while limiting systemic cytokine leakage [159, 160]. This spatial safety problem is further complicated by the temporal dimension of nanomaterial retention. Repeated administration may amplify immune sensitization, accelerated clearance or unintended activation of distal lymphoid organs [161], while persistent intranodal residence may become a local source of chronic nodal inflammation and tissue remodeling. In breast cancer-related SLN tattooing, melanin-loaded PLGA nanoparticles remained detectable in tattooed LNs for up to 16 weeks and induced foreign-body-like granulomatous inflammation, multinucleated giant cell formation and macrophage damage [162]. Such persistent foreign-body reactions are relevant not only to local inflammatory toxicity, but also to potential fibrosis-like remodeling, altered lymphatic drainage and disruption of nodal immune homeostasis. PLGA-encapsulated carbon nano/microparticles also produced material-parameter-dependent foreign-body inflammatory reactions in porcine LNs. However, inflammation decreased over time and no fibrotic tissue was detected, suggesting that nodal risk is governed by particle design, degradation and clearance rather than retention alone [163]. Material composition further modifies this risk: cadmium-free CuInS_2_/ZnS quantum dots induced acute axillary LN inflammation only at a higher dose than Cd-containing QDs [164], whereas CdSe/ZnS quantum dots remained detectable in mouse LNs for up to two years with spectral changes suggestive of in vivo transformation [165]. Together, these findings indicate that persistent nodal retention is not inherently unsafe, but its consequences are highly dependent on material composition, degradation behavior, inflammatory persistence and clearance kinetics. Importantly, chronic intranodal inflammation may perturb lymphatic drainage, immune cell trafficking and antigen presentation, thereby increasing the risk of lymphatic dysfunction and aberrant immune activation, including autoimmune-like responses in susceptible hosts. Therefore, future TDLN-targeted nanomedicines should be evaluated not only for acute systemic toxicity, but also for long-term nodal inflammation, fibrosis-like remodeling, lymphatic function and immune tolerance after repeated or prolonged nodal exposure.

**Fig. 7:**
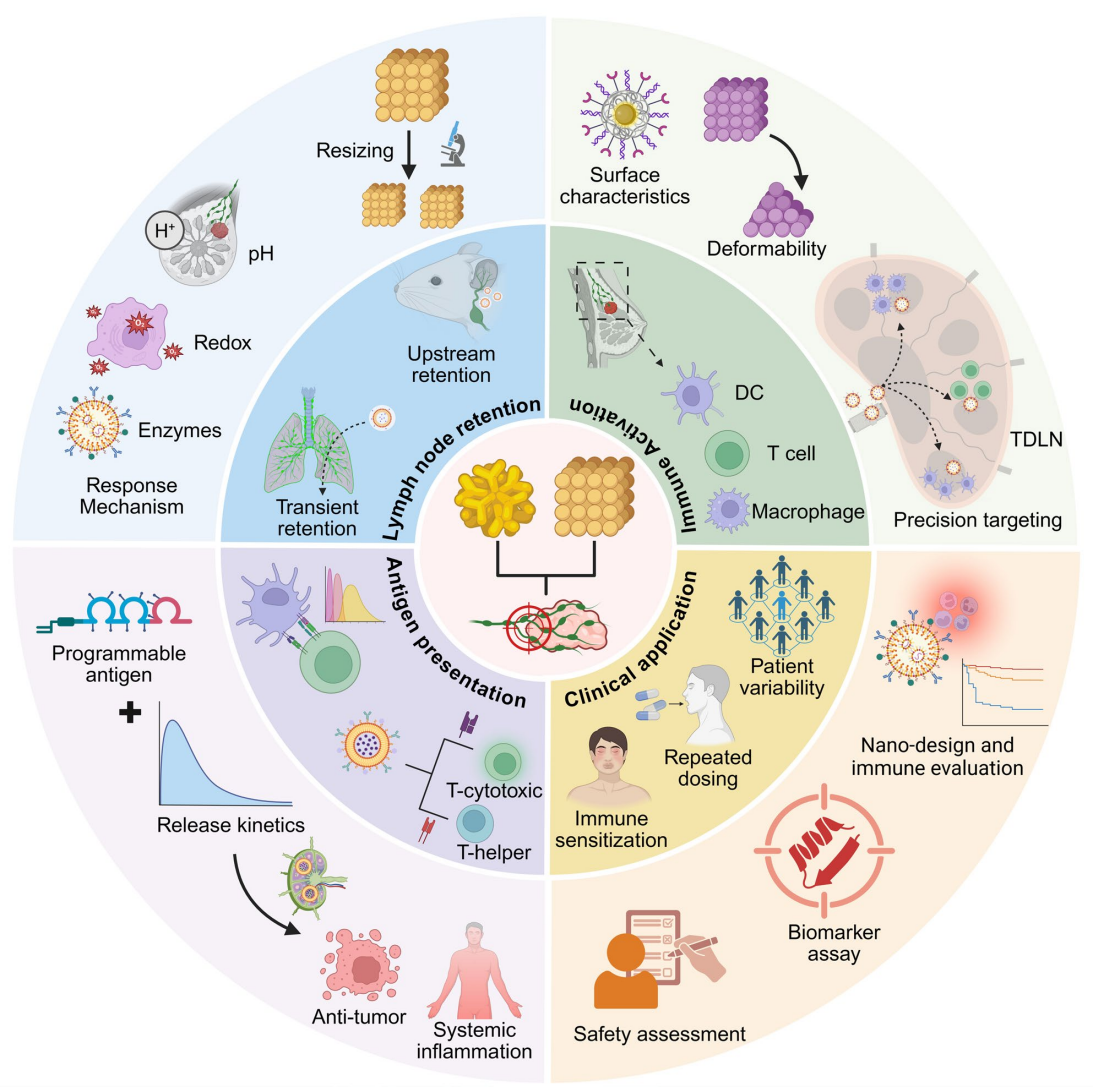
Challenges and future directions in nanomaterial design for targeting TDLNs and SLNs in cancer immunotherapy. Despite significant progress in targeting TDLNs and SLNs with nanomaterials, several biological and practical barriers remain in clinical translation. The figure first highlights the physicochemical properties of nanomaterials, including particle size, surface characteristics, and deformability, which play crucial roles in lymphatic transport and interactions with immune cells. Nanomaterials can promote antigen presentation and T cell priming by targeting DCs, macrophages, and T cells, thereby enhancing tumor immune responses. The figure also emphasizes how optimizing nanomaterial design can address challenges related to lymph node retention and immune activation, particularly in the precise targeting and delivery to TDLNs. Additionally, the figure explores the clinical potential of these nanomaterials, focusing on how stage-adaptive nanomaterials can overcome immune evasion issues and improve therapeutic outcomes by optimizing patient variability and immune competence assessments, while minimizing immune-related adverse events and long-term toxicity

A major barrier to clinical translation is the lack of predictive frameworks that account for interpatient variability in lymphatic architecture, nodal immune state and metastatic burden. Murine TDLN-targeting studies have established important proof of concept, showing that intradermally delivered adjuvanted polymeric nanoparticles or vaccine formulations can accumulate in TDLNs, activate DCs and enhance antitumor immunity under controlled experimental conditions [26, 27]. However, preclinical efficacy in homogeneous murine models often fails to capture the dynamic and heterogeneous nature of human TDLNs, leading to inconsistent therapeutic outcomes [166]. This limitation becomes more evident as the model system moves closer to human tissue scale. In a swine large-animal model, 50 nm expansile polymer nanoparticles, but not 100 nm particles, migrated through lymphatics to regional lymph nodes and delivered paclitaxel to subcapsular nodal spaces, indicating that particle size, injection route and lymphatic anatomy can critically constrain nodal drug delivery [167]. Early clinical data further highlight the difficulty of translating nodal immune accumulation into durable patient benefit. In a phase 1 study registered as JMA-IIA00346 evaluated New York esophageal squamous cell carcinoma 1-specific TCR-engineered T (TCR-T) cell therapy combined with a lymph node-targeting pullulan nanogel long-peptide vaccine for advanced soft tissue sarcoma, paired mouse experiments showed increased TCR-T cell accumulation in draining lymph nodes and tumors, whereas durable tumor shrinkage and long-term TCR-T cell persistence were reported in only one patient [168]. Together, these cross-species comparisons highlight that parameters such as particle size, lymphatic transport dynamics and nodal immune heterogeneity are scaled differently across model systems, and that effective TDLN targeting in murine models does not directly translate into predictable clinical efficacy. Progress in this area will require TDLN-informed design and patient stratification frameworks that treat nodal accessibility, immune competence and suppressive features as input variables rather than post hoc observations, supported by imaging, nodal biomarkers and functional immune readouts to link nanomaterial design with nodal immune programming and clinical response. (Fig. 7).

## Conclusion

TDLNs have traditionally been approached as indicators of metastatic spread or targets for surgical management rather than as active participants in antitumor immunity. The studies discussed in this review support a growing shift away from this view, demonstrating that TDLNs are dynamic immune organs whose functional state can be deliberately modulated. Nanotechnology offers a means to access these lymphatic hubs with a level of spatial and temporal control that is difficult to achieve using conventional systemic therapies.

Despite this progress, several challenges must be addressed before TDLN-targeted nanomedicine can be reliably translated into clinical benefit. Efficient lymphatic delivery does not necessarily result in effective immune engagement, underscoring the need to move beyond nodal accumulation as a primary performance metric. Greater emphasis is required on controlling intranodal localization, cellular routing and the timing of immune activation, as well as on ensuring that local immunomodulation does not lead to unintended systemic toxicity or long-term disruption of lymph node homeostasis. In parallel, variability in lymphatic architecture and nodal immune states across tumor types and patient populations continues to complicate prediction of therapeutic response. Addressing these limitations will require a transition from material-centered optimization toward framework-driven design strategies that integrate lymph node biology, immune dynamics and safety considerations as core constraints. By aligning nanoplatform engineering with the immunological logic of TDLNs, future approaches may enable more precise coordination between local tumor control and durable systemic antitumor immunity.

## Data Availability

No datasets were generated or analysed during the current study.

## Abbreviations

APCs: Antigen-presenting cells
ApoE3: Apolipoprotein E3
APS: 3-aminopropyl-triethoxysilane
CCZC: CaO_2_@Curcumin@ZIF-Cu
cdGMP: Cyclic di-GMP
CHOL@GEM-NBs: Gemcitabine-loaded nanobubbles with a cholesterol component shell
CpG: Cytosine guanine dinucleotide
C-RNA-NPs: Chemotherapy-induced tumor RNA nanoparticles
CXCR4: CXC chemokine receptor type 4
DAMP: Damage-associated molecular pattern
DCs: Dendritic cells
DOX: Doxorubicin
E. coli: Escherichia coli
HA: Hyaluronic acid
HCC: Hepatocellular carcinoma
HDL: High-density lipoprotein
HMCS-PVP: Hollow mesoporous carbon spheres functionalized with pvp
ICD: Immunogenic cell death
ICIs: Immune checkpoint inhibitors
IDO: 2,3-dioxygenase-1
iNKT: Invariant natural killer T
LAG-3: Lymphocyte activation gene 3
LNPs: Lipid nanoparticles
LNs: Lymph nodes
MHC: Major histocompatibility complex
MPLA: Monophosphoryl lipid
NIR: Near-infrared
NK: Natural killer
NPC1L1: Niemann-pick c1-like 1
NVTRAIL: Engineered cellular nanovesicles displaying membrane-bound trail
OMVs: Outer membrane vesicles
OVA: Ovalbumin
ox-MWCNTs: Acid-functionalized multiwalled-carbon nanotubes
PBAE: Poly(β-amino ester)
PCL: Poly(ε-caprolactone)
PEG: Poly(ethylene glycol)
PEG-PLL–PLLeu: Peg-poly (l-lysine)-poly (l-leucine)
PEI: Polyethylenimine
PET: Positron emission tomography
PFCE: Perfluoro-15-crown-5-ether
PLA: Poly(lactic acid)
PLGA: Poly(lactic-co-glycolic acid)
PRINT: Particle replication in nonwetting templates
PTT: Photothermal therapy
R-DOTAP: R-1, 2-dioleoyl-3-trimethyl-ammonium-propane
RT: Radiotherapy
SLNs: Sentinel lymph nodes
SPIONs: Superparamagnetic iron oxide nanoparticles
TAAs: Tumor-associated antigens
TCL: Tumor cell lysates
TCR: T cell receptor
TCR-T: T cell receptor-engineered T
TDLNs: Tumor-draining lymph nodes
Tfh: Follicular helper T
TLR7: Toll-like receptor 7
TPP: Tripolyphosphate
Treg: Regulatory T cell
V-scVLPs: Virus-like silicon nanoparticles
